# The elevated transcription of ADAM19 by the oncohistone H2BE76K contributes to oncogenic properties in breast cancer

**DOI:** 10.1016/j.jbc.2021.100374

**Published:** 2021-02-04

**Authors:** Tze Zhen Evangeline Kang, Lina Zhu, Du Yang, Dongbo Ding, Xiaoxuan Zhu, Yi Ching Esther Wan, Jiaxian Liu, Saravanan Ramakrishnan, Landon Long Chan, Siu Yuen Chan, Xin Wang, Haiyun Gan, Junhong Han, Toyotaka Ishibashi, Qing Li, Kui Ming Chan

**Affiliations:** 1 Department of Biomedical Sciences, City University of Hong Kong, Hong Kong, China; 2 Key Laboratory of Biochip Technology, Biotech and Health Centre, Shenzhen Research Institute of City University of Hong Kong, Shenzhen, China; 3 State Key Laboratory of Protein and Plant Gene Research, School of Life Sciences and Peking-Tsinghua Center for Life Sciences, Peking University, Peking, China; 4 Division of Life Science, Hong Kong University of Science and Technology, Hong Kong, China; 5 Department of Oncology, Princess Margaret Hospital, Hong Kong, China; 6 Department of Paediatrics and Adolescent Medicine, The University of Hong Kong, Hong Kong, China; 7 Guangdong Provincial Key Laboratory of Synthetic Genomics, CAS Key Laboratory of Quantitative Engineering Biology and Shenzhen Key Laboratory of Synthetic Genomics, Shenzhen Institute of Synthetic Biology, Shenzhen Institutes of Advanced Technology, Chinese Academy of Sciences, Shenzhen, China; 8 State Key Laboratory of Biotherapy and Cancer Center, West China Hospital, West China Medical School, Sichuan University, Sichuan, China

**Keywords:** oncohistone, breast cancer, ADAM, transcription, epigenetics, *ADAM19*, a disintegrin and metalloproteinase-domain-containing protein 19, BH, Benjamini–Hochberg, BRCA, breast invasive carcinoma, DIPG, diffuse intrinsic pontine glioma, DRB, 5,6-dichlorobenzimidazole1-β-D-ribofuranoside, EMT, epithelial–mesenchymal transition, GO, gene ontology, GSEA, gene set enrichment analysis, HDR, homology-directed repair, IP, immunoprecipitation, KI, knock-in, NT, nontargeting, P-TEFb, positive transcription elongation factor-b, PDAC, pancreatic ductal adenocarcinoma, PEI, polyethylenimine, PTM, posttranslational modification, shRNA, short hairpin RNA, TSS, transcription start site, WT, wild-type

## Abstract

The recent discovery of the cancer-associated E76K mutation in histone H2B (H2BE76-to-K) in several types of cancers revealed a new class of oncohistone. H2BE76K weakens the stability of histone octamers, alters gene expression, and promotes colony formation. However, the mechanism linking the H2BE76K mutation to cancer development remains largely unknown. In this study, we knock in the H2BE76K mutation in MDA-MB-231 breast cancer cells using CRISPR/Cas9 and show that the E76K mutant histone H2B preferentially localizes to genic regions. Interestingly, genes upregulated in the H2BE76K mutant cells are enriched for the E76K mutant H2B and are involved in cell adhesion and proliferation pathways. We focused on one H2BE76K target gene, *ADAM19* (a disintegrin and metalloproteinase-domain-containing protein 19), a gene highly expressed in various human cancers including breast invasive carcinoma, and demonstrate that H2BE76K directly promotes *ADAM19* transcription by facilitating efficient transcription along the gene body. *ADAM19* depletion reduced the colony formation ability of the H2BE76K mutant cells, whereas wild-type MDA-MB-231 cells overexpressing *ADAM19* mimics the colony formation phenotype of the H2BE76K mutant cells. Collectively, our data demonstrate the mechanism by which H2BE76K deregulates the expression of genes that control oncogenic properties through a combined effect of its specific genomic localization and nucleosome destabilization effect.

Histones are nuclear proteins required for the packaging of genomic DNA in the form of chromatin. Two copies of each of the core histones H2A, H2B, H3, and H4 with ∼147 bp of DNA form a nucleosome, the basic unit of chromatin (1). The compaction of DNA into the nucleosome, however, creates major obstacles to gene regulatory processes, such as transcription and DNA replication. Nucleosome dynamics, regulated in part by histone posttranslational modifications (PTMs) (2), thus play a major role in modulating gene activity by controlling DNA accessibility. Misregulation of histone PTMs has been extensively linked to cancer (3) and was most recently implicated in cancers containing mutations in genes encoding histone H3 (4). These include the H3K27M and H3G34R/V mutations found in pediatric gliomas (5, 6) and the H3G34W/L and H3K36M mutations found in sarcomas (7). We and others have previously demonstrated the effect of the H3K27-to-M mutation in H3K27 di- and tri-methylation as well as transcriptional misregulation in diffuse intrinsic pontine glioma (DIPG) (8, 9). The H3G34V/R/W/L mutations affect the methylation of lysine 36 in *cis* while the H3K36M mutation exhibits a dominant negative effect and inhibits the methylation of lysine 36 in *trans* (9, 10, 11). Notably, these cancer-associated H3 mutations are all located at or near key lysine residues that are posttranslationally modified and thus affect key regulatory PTMs, subsequently driving oncogenesis through changes in the epigenetic landscape and gene expression profiles.

To address whether additional histone mutations exist in other malignancies, we searched for missense mutations that occur in histone encoding genes in cancer patient samples through the cBioPortal database (12). We identified a couple of novel cancer-associated mutations in genes encoding histone H2B, including the H2BG53D mutation in pancreatic ductal adenocarcinoma (PDAC) we recently reported (13, 14), and mutations of the Glu76 (E76) residue of histone H2B. Consistent with recent reports (15, 16), we found the E76 residue to be the most frequently mutated H2B residue in various cancers including breast and lung carcinoma, which contained either Glu76Lys (E76K) or Glu76Gln (E76Q) missense mutations in genes encoding H2B (Table S1). Interestingly, the ectopic overexpression of H2BE76K has been previously shown to enhance colony formation ability in mouse fibroblast and human epithelial cells, suggesting that H2BE76K can confer oncogenic properties (16, 17). Within the nucleosome, the H2BE76 residue (equivalent to E73 in *Xenopus laevis*) interacts directly with the R92 residue of histone H4 through hydrogen bonding at the H2A-H2B dimer–(H3-H4)_2_ tetramer interface (1). Recent studies showed that the abolishment of this interaction by H2BE76K destabilizes the nucleosome (17). Unlike the H3K27M and H3K36M mutations, which directly alter global levels H3K27 and H3K36 methylation, respectively, H2BE76K does not occur on a posttranslationally modified site and is therefore unlikely to directly affect PTMs (18). Instead, the H2BE76K mutation has been suggested to promote gene expression through its nucleosome destabilizing effect (16). However, the complete picture of how H2BE76K induces transcriptional misregulation remains unclear, as H2BE76K expression has been shown to result in both gene activation and repression (16). The mechanism of how H2BE76K mediates specific transcriptional changes to promote oncogenic phenotypes also remains to be answered.

The average tumor allele frequency of the H2BE76K mutation in tumors (∼20%) suggests that it is an acquired subclonal mutation rather than a germline mutation and is likely to cooperate with other driver mutations in later stages of cancer progression (15, 16) (Table S1). Supporting this notion, the expression of H2BE76K alone has been shown to have no effect on the normal mammary epithelial cell line MCF10A, but could enhance colony formation ability in the presence of the oncogenic PI3KCA-H1047R mutant (16). To dissect the molecular mechanism by which H2BE76K promotes breast cancer development, we knock in the H2BE76K mutation in the MDA-MB-231 breast cancer cell line. Using CUT&RUN, we mapped the genome-wide localization of the H2BE76K mutant histone and found that H2BE76K preferentially localizes on gene bodies. We further identify the H2BE76K-enriched gene *ADAM19* as a direct target linking H2BE76K to oncogenic phenotypes. Collectively, these findings illustrate the effects of the H2BE76K mutation in transcription misregulation and breast cancer development.

## Results

### The H2BE76K mutation destabilizes H2B-H3/H4 interactions

**Figure 1:**
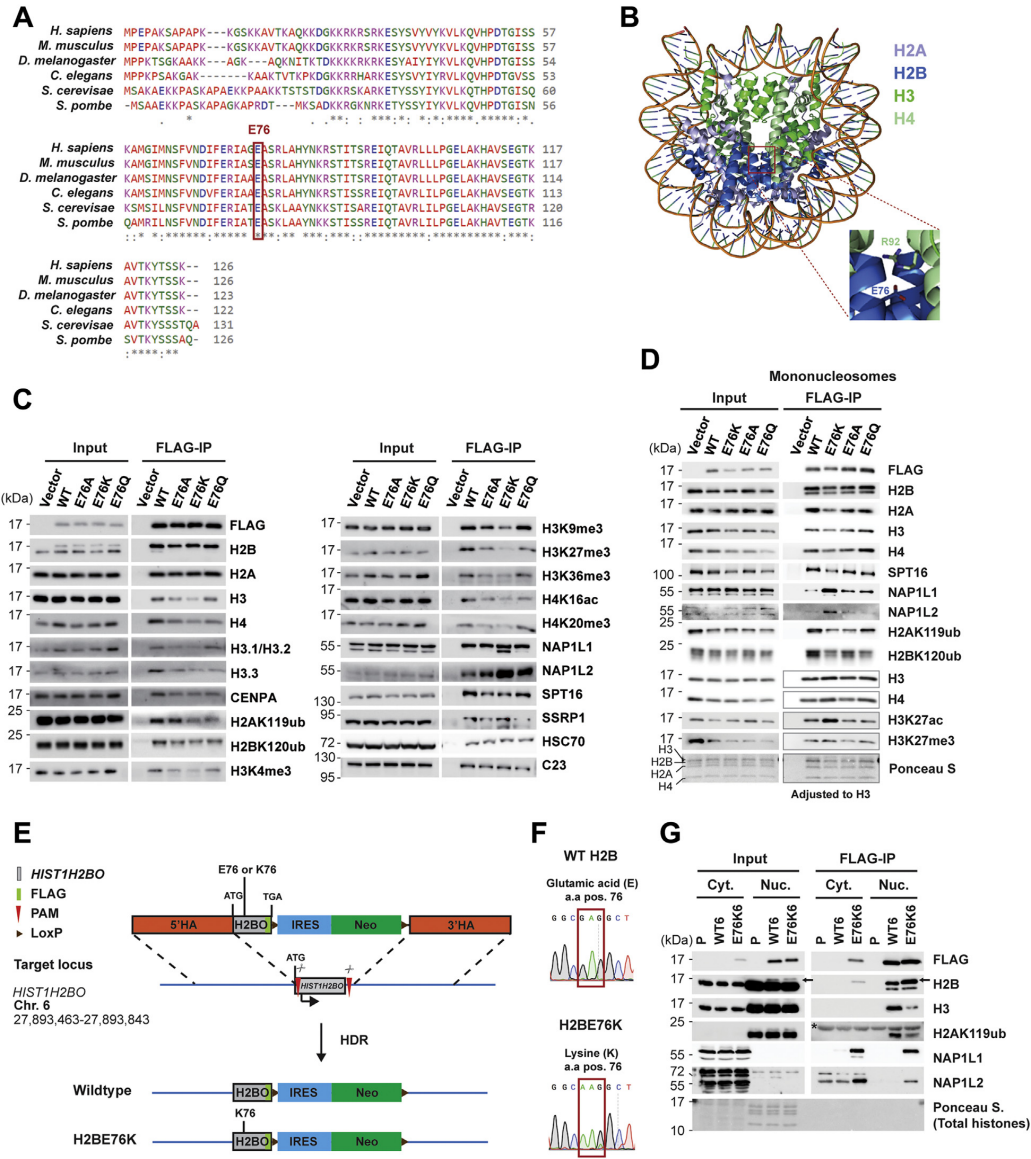
**H2BE76K mutation alters nucleosome stability, H2B-histone chaperones interactions, and histone modifications in *cis*.***A*, H2B glutamic acid at 76 is evolutionary conserved from yeast to higher organisms. Multiple sequence alignment of H2B proteins from *Homo sapiens* (H2B1C/E/F/G/I), *Mus musculus* (H2B1C/E/G), *Drosophila melanogaster* (H2B), *Caenorhabditis elegans* (H2B 1), *Saccharomyces cerevisiae* (H2B.1), and *Schizosaccharomyces pombe* (H2B.1). Glutamic acid at position 76 of the human H2B protein is highlighted in *red rectangle*. *B*, H2BE76 residue is located at the center of the histone octamer. Structural analysis of human nucleosome with core histones H2A, H2B, H3, and H4 labeled in *indicated colors*. *C*, H2BE76K mutation weakens H2B-H3/H4 interactions and enhances H2B association with NAP1L proteins. 293T cells expressing FLAG-tagged WT H2B, E76A, E76K, and E76Q H2B mutants were subjected to FLAG-IP. Input and IP samples were analyzed by immunoblotting using indicated antibodies. *D*, H2BE76K-containing nucleosomes showed increased H3K27ac and reduced H2AK119ub. Immunoblot analysis of mononucleosomes immunoprecipitated with αFLAG antibody from cross-linked 293T cells expressing FLAG-tagged WT H2B and H2B E76K/A/Q mutants. To compensate for the decreased interaction between E76 mutants and H3/H4, IP samples were adjusted to equalized amount of H3 between samples. *E*, generation of FLAG-H2B WT/E76K knock-in (KI) MDA-MB-231 cell lines. Schematic diagram of the CRISPR/Cas9-mediated HDR targeting strategy for the *HIST1H2BO* locus. The double-stranded break induced in the target locus was repaired precisely through HDR between the donor plasmid (includes FLAG-*HIST1H2BO*, IRES, Neomycin resistance sequence, LoxP sites, and homology arms) and the target locus. PAM, protospacer adjacent motif. *F*, sanger sequencing confirmed the H2BE76K mutation in the *HIST1H2BO* locus. Sequencing chromatograms of a WT and H2BE76K mutant clone. *G*, H2BE76K shows chromatin localization defect and increased binding to NAP1L proteins. Parental and KI MDA-MB-231 cell extracts were separated into cytoplasmic and mononucleosome fractions and immunoprecipitated with αFLAG antibody. Mononucleosomes were purified from native chromatin. Input and IP samples were analyzed by immunoblotting using indicated antibodies. The *arrow* points to FLAG-tagged WT H2B/H2BE76K. ∗ indicates an unspecific band.

The E76 residue of histone H2B is evolutionarily conserved among yeast and higher eukaryotes (Fig. 1*A*) and is located at the H2B-H4 interface in the center of the histone octamer (Fig. 1*B*). Recent studies have shown that the H2BE76K mutation destabilizes the nucleosome by disrupting H2B-H4 interactions (16, 17). In addition to H2BE76K’s effect on nucleosome stability, we asked whether the mutation might also affect histone modifications and nucleosome–histone chaperone interactions. To address this, FLAG-tagged H2BE76 mutants were expressed in 293T cells followed by FLAG immunoprecipitation (IP). FLAG-IP results confirmed the negative effect of the H2BE76K mutation on H2B-H3/H4 interaction (Fig. 1*C*). The substitution of H2BE76 to K and Q, as well as to the neutrally charged alanine (A), reduced H2B association with H3 and H4, with the E76K mutant showing the strongest effect. Intriguingly, we also found that the H2BE76K, but not the H2BE76Q mutant, displayed elevated interaction with both the H2A-H2B chaperones nucleosome assembly protein 1-Like 1 and 2 (NAP1L1 and NAP1L2) and reduced interaction with the H2A-H2B chaperone FACT subunit SPT16. While the presence of the mutant H2BE76K did not affect global histone modification levels in transfected 293T cells, the H2BE76K mutation led to a reduced association with H2AK119 ubiquitination (H2AK119ub), a repressive histone mark (19), and H2BK120 ubiquitination, a histone mark associated with active transcription elongation (20). We confirmed the reduced H2AK119ub and H2BK120ub levels in fixed mononucleosomes containing H2BE76 mutants (Fig. 1*D*). Interestingly we also found increased H3K27 acetylation (H3K27ac) levels specifically on H2BE76K-containing nucleosomes. While both H2BE76K and H2BE76Q mutations reduced H2AK119ub and H2BK120ub levels in *cis*, the two mutations appear to be functionally distinct as H2BE76K, but not the H2BE76Q mutant, protein differentially binds to histone chaperones.

### Generation of H2BE76K knock-in breast cancer cells by CRISPR/Cas9

We reasoned that since the H2BE76K mutation is an acquired subclonal mutation, normal nontransformed cells might not acquire oncogenic phenotypes when the H2BE76K mutant histone is expressed at a physiological level. To study the effect of H2BE76K mutation in cancer development, we generated knock-in (KI) mutant cell lines using the MDA-MB-231 breast cancer cell line. The CRISPR/Cas9 system was employed to introduce the H2BE76K mutation into an H2B encoding gene to express the H2BE76K mutant at a physiological level mimicking patient conditions (Fig. 1, *E* and *F*). To achieve this, we substituted one of the 17 somatic H2B encoding genes, *HIST1H2BO*, with a wild-type (WT) or E76K mutated H2B through CRISPR/Cas9-mediated homology-directed repair (HDR). We chose to target *HIST1H2BO* since 1) the H2BE76K mutation was found in this H2B gene in breast cancer (Table S1) and 2) to minimize CRISPR/Cas9 off-target effects, as the sgRNAs targeting this H2B gene had the highest predicted specificity scores. We tagged both the WT and E76K mutant H2B with a FLAG-tag at the C-terminal tail for the assessment of expression levels and the mapping of the genomic distribution of the H2BE76K mutant histones. The targeted editing was confirmed through PCR genotyping with no effects on the top predicted off-target sites and on other H2B genes with high sequence similarity to *HIST1H2BO* (Fig. S1, *A*–*C*, Fig. S2, *A* and *B*). Herein, KI cell lines expressing FLAG-WT H2B and FLAG-H2BE76K are referred to as WT and H2BE76K cells, respectively. Global histone modifications levels were unaffected in the mutant cell lines carrying either the WT or E76K mutant H2B (Fig. S3). Cell fractionation assays revealed a small portion of H2BE76K that failed to deposit on the chromatin and remained in the cytosolic fraction; however, the majority of the E76K mutant H2B was still successfully incorporated into the chromatin (Fig. 1*G*, Fig. S4). We subsequently performed FLAG-IP using the cytoplasmic and mononucleosomes fractions and found that both cytosolic and chromatin-bound H2BE76K protein interacts with NAP1L1 and NAP1L2 (Fig. 1*G*), similar to the increased H2BE76K-NAP1L1/NAP1L2 interactions observed in 293T cells (Fig. 1, *C* and *D*). The H2BE76K-containing nucleosomes additionally displayed decreased H2AK119ub, in agreement with the results of the IP experiments performed with 293T cells (Fig. 1*C*).

### Preferential localization of E76K mutant H2B to gene bodies

**Figure 2:**
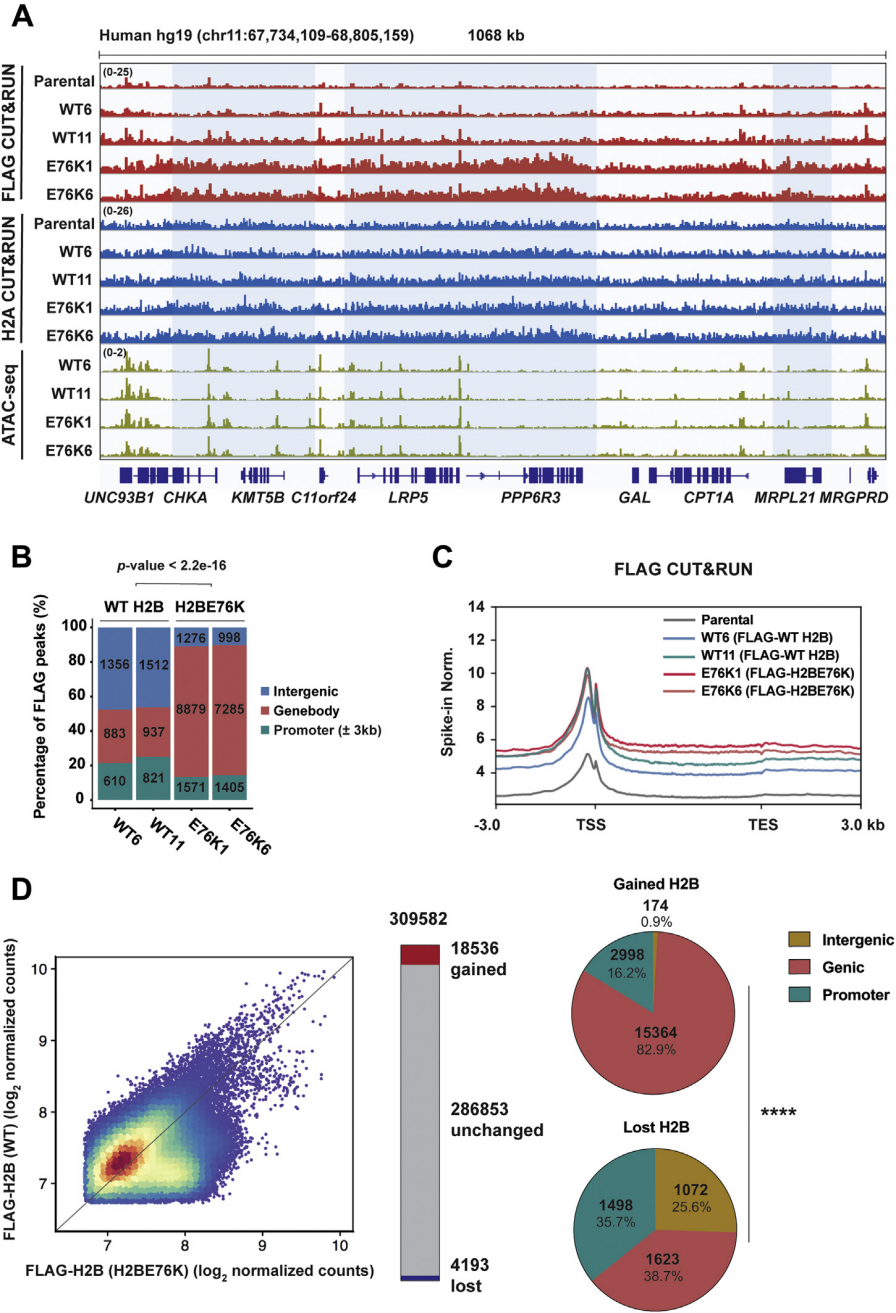
**Genome-wide profiling of H2BE76K.***A*, a snapshot of FLAG-H2BE76K-enriched genes. Integrative Genomics Viewer (IGV) tracks of FLAG CUT&RUN, H2A CUT&RUN, and ATAC-seq in chromosome 11. Regions highlighted in *blue* show FLAG-H2BE76K-enriched genes. *B*, H2BE76K peaks are enriched in gene body regions. Distribution of FLAG-WT H2B and FLAG-H2BE76K CUT&RUN peaks in promoter (±3 kb), gene body, and intergenic regions. Number of FLAG peaks associated with each genomic region are indicated on the *bars*. *p*-value was calculated using the *Chi*-square test. *C*, FLAG CUT&RUN profile on gene bodies. Metaplot showing average FLAG CUT&RUN-seq signals in parental MDA-MB-231, WT, and E76K cell lines from 3 kb upstream of transcript start site (TSS) to 3 kb downstream of transcription end site (TES). Data are represented as yeast spike-in normalized reads. *D*, genome-wide distribution of FLAG-H2B proteins in WT and H2BE76K mutant cell lines. FLAG-H2B enrichment in WT and H2BE76K was compared by analyzing gains and losses of FLAG-H2B reads in 10 kb bins. *Left*, scatterplot shows the changes in FLAG-H2B enrichment in H2BE76K mutant cells. Bins with above-median averages were analyzed for gains and losses of fold change 1.5. *Middle*, barplot shows the gained (*red*) and lost (*blue*) FLAG-H2BE76K relative to FLAG-WT H2B. *Bottom*, pie charts show the proportion of gained H2BE76K (*middle*) and lost H2BE76K (*right*) 10 kb bins in intergenic, genic, and promoter regions. *p*-value was calculated using the Chi-square test (∗∗∗∗*p* ≤ 0.0001).

While a previous study has shown the transcriptional changes that accompanied the ectopic overexpression of H2BE76K, the exact mechanism underlying H2BE76K’s effect on gene expression remains unclear (16). To understand H2BE76K’s direct effect on gene expression, we performed CUT&RUN (21) to map the genomic localization of the FLAG-tagged WT H2B and H2BE76K mutant histones in WT and H2BE76K cell lines, respectively (Fig. 2*A*, Fig. S5). Histone H2A CUT&RUN was performed to assess nucleosomal occupancy and additionally acts as an internal control for the FLAG CUT&RUN (Fig. S5A). Intriguingly, we found that the genomic distribution of FLAG-H2BE76K was markedly different compared with that of FLAG-WT H2B. While the major portion of FLAG-WT H2B peaks were localized in intergenic regions, more than 70% of FLAG-H2BE76K peaks are located in genic regions (Fig. 2*B*). Profiles of average FLAG CUT&RUN signals show that FLAG-H2BE76K is specifically enriched on gene body regions, downstream of the transcription start site (TSS) (Fig. 2*C*). The increased genic FLAG-H2BE76K enrichment was not attributed to nucleosomal occupancy changes, as the average H2A CUT&RUN signals were comparable between the WT and H2BE76K mutant cells (Fig. S5).

A previous report on H2BE76K’s effect on chromatin accessibility showed that ectopic H2BE76K overexpression promoted chromatin accessibility and gene expression in MCF10a cells (16). Interestingly, however, our genome-wide chromatin accessibility using ATAC-seq (Assay for transposase-accessible chromatin sequencing) (22) showed that H2BE76K-enriched genes, as exemplified at the *CHKA*, *KMT5B*, *LRP5*, *PPP6R3*, and *MRPL21* loci, did not display apparent chromatin accessibility changes in our H2BE76K mutant cells (Fig. 2*A*). We further performed differential occupancy analysis on genes and found significant FLAG-H2BE76K enrichment (Benjamini–Hochberg (BH) adjusted *p* < 0.05, log_2_ fold change (log_2_FC) > 0.5) in more than 2000 genes (Fig. S5C). We validated the FLAG CUT&RUN results by chromatin immunoprecipitation (ChIP)-qPCR and confirmed H2BE76K enrichment patterns on selected genes in chromosome 11 highlighted in Figure 2*A* (Fig. S6). Analysis of genome-wide differential distribution patterns between WT H2B and H2BE76K further revealed the specific gain of H2BE76K enrichment in promoter (16.2%) and genic (82.9%) regions, whereas the lost H2B occurred equally across promoter (35.7%), genic (38.7%), and intergenic (25.7%) regions (Fig. 2*D*). Collectively, these results show that H2BE76K is redistributed to and preferentially localizes in genic regions.

### H2BE76K enrichment correlates with elevated gene expression

**Figure 3:**
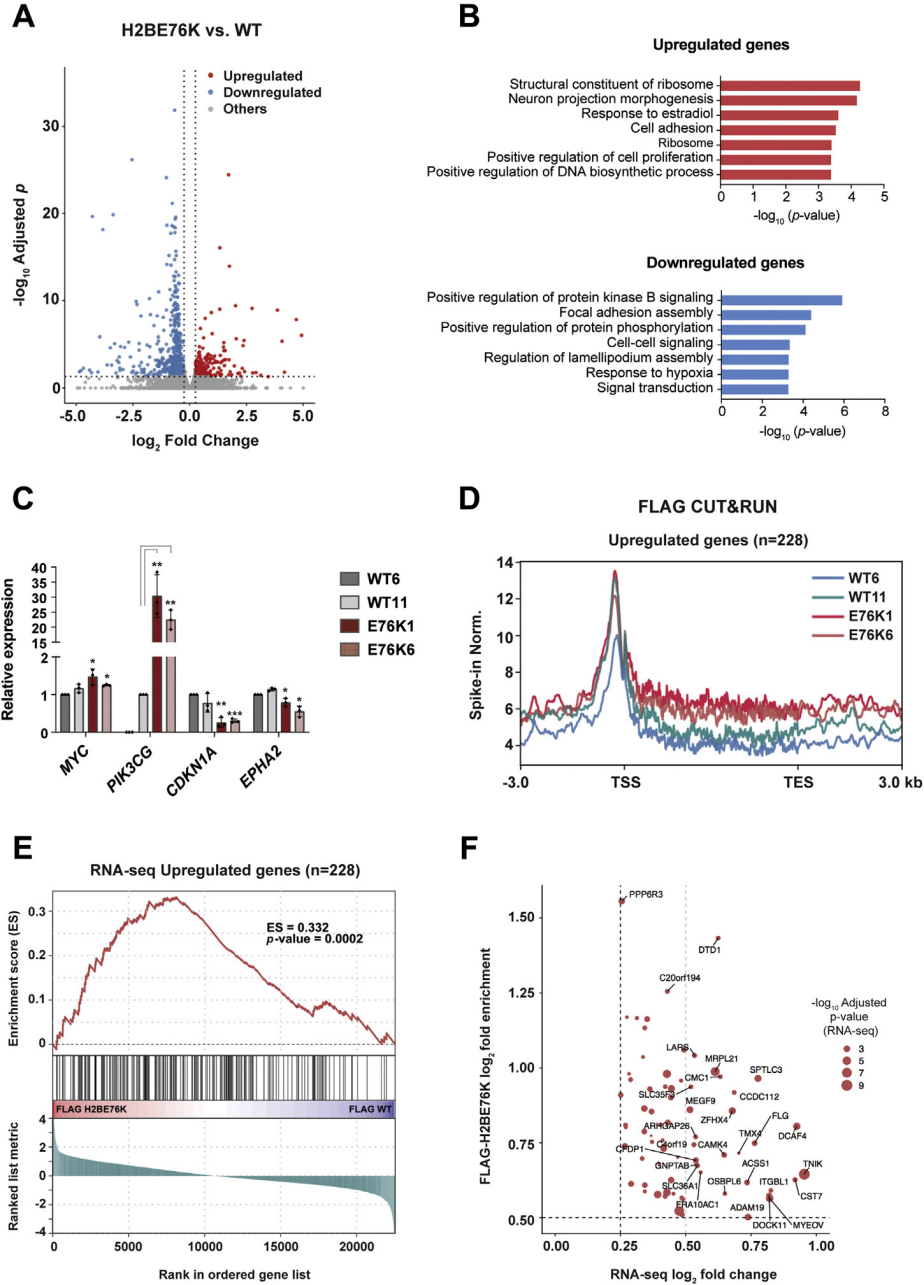
**H2BE76K mutation induces gene expression changes in breast cancer cells.***A*, Volcano plot showing the differentially expressed genes between WT and H2BE76K mutant cell lines (BH adjusted *p* < 0.05, absolute log_2_FC > 0.25) *B*, the top gene ontology (GO) terms enriched among genes that are significantly upregulated and downregulated in the E76K cells. *p*-values were calculated by hypergeometric test. *C*, mRNA expression of *MYC*, *PIK3CG*, *CDKN1A*, and *EPHA2* in WT and E76K cell lines. *PIK3CG* is not expressed in the WT6 cell line. Results from three independent RT-qPCR experiments are shown (mean ± SD, ∗*p* ≤ 0.05, ∗∗*p* ≤ 0.01, ∗∗∗*p* ≤ 0.001, ∗∗∗∗*p* ≤ 0.0001). *p*-values were calculated using one-sided *t*-test between each E76K sample and WT6 unless indicated otherwise. *D*, gene bodies of upregulated genes in the E76K cells are enriched with H2BE76K. Metaplots showing average FLAG CUT&RUN-seq signals in parental MDA-MB-231, WT, and E76K cell lines from 3 kb upstream of TSS to 3 kb downstream of TES. Data are represented as yeast spike-in normalized reads. *E*, upregulated genes in the H2BE76K mutant cells are enriched with H2BE76K mutant histone. GSEA was performed using genes upregulated in E76K cells. Vertical lines show each upregulated gene. Gene list was ranked by differential H2BE76K occupancy between H2BE76K mutant and WT cells. *F*, E76K-upregulated genes with significant H2BE76K occupancy (BH adjusted *p* < 0.05, log_2_ fold enrichment >0.5) are shown. The gene list is found in Table S9.

To further investigate the effect of the H2BE76K mutation on gene expression, we analyzed the transcriptomes of the KI cell lines using RNA-sequencing (RNA-seq). The H2BE76K cell lines exhibited a distinct gene expression profile with 228 significantly upregulated and 493 downregulated genes (BH adjusted *p* < 0.05, absolute log_2_FC > 0.25) from two independent experiments comparing two H2BE76K KI clones and two isogenic WT clones (Fig. 3*A*, Fig. S7). Importantly, we found that some cancer-associated pathways such as cell adhesion and cell proliferation were significantly enriched in genes showing upregulation by gene ontology (GO) analysis. In contrast, cancer-related pathways such as focal adhesion assembly, cell signaling as well as response to hypoxia were also significantly enriched in downregulated genes (Fig. 3*B*). We performed RT-qPCR and confirmed the differential expression of several cancer driver genes (23): increased mRNA expression of oncogenes *MYC* and *PIK3CG*, as well as decreased expression of tumor suppressor genes *CDKN1A* and *EPHA2*, was observed in the H2BE76K mutant cells (Fig. 3*C*). To investigate the direct role of H2BE76K in gene misregulation, we analyzed H2BE76K’s genomic localization in relation to gene expression changes (Fig. 3*D*, Fig. S8, *A* and *B*). Intriguingly, we observed that genes upregulated in H2BE76K mutant cells exhibited increased H2BE76K enrichment (enrichment over FLAG-WT H2B) (Fig. 3*D*). As represented by H2A CUT&RUN, total nucleosomal occupancy on the upregulated genes was comparable between WT and H2BE76K mutant cells (Fig. S9, *C*–*E*). More importantly, gene set enrichment analysis (GSEA) revealed a positive correlation between upregulated genes and H2BE76K enrichment, suggesting a relationship between H2BE76K enrichment and increased transcription (Fig. 3*E*). Figure 3*F* shows the 78 upregulated genes with the most significant H2BE76K enrichment (Table S2).

### ADAM19 promotes colony formation ability in H2BE76K mutant cells

**Figure 4:**
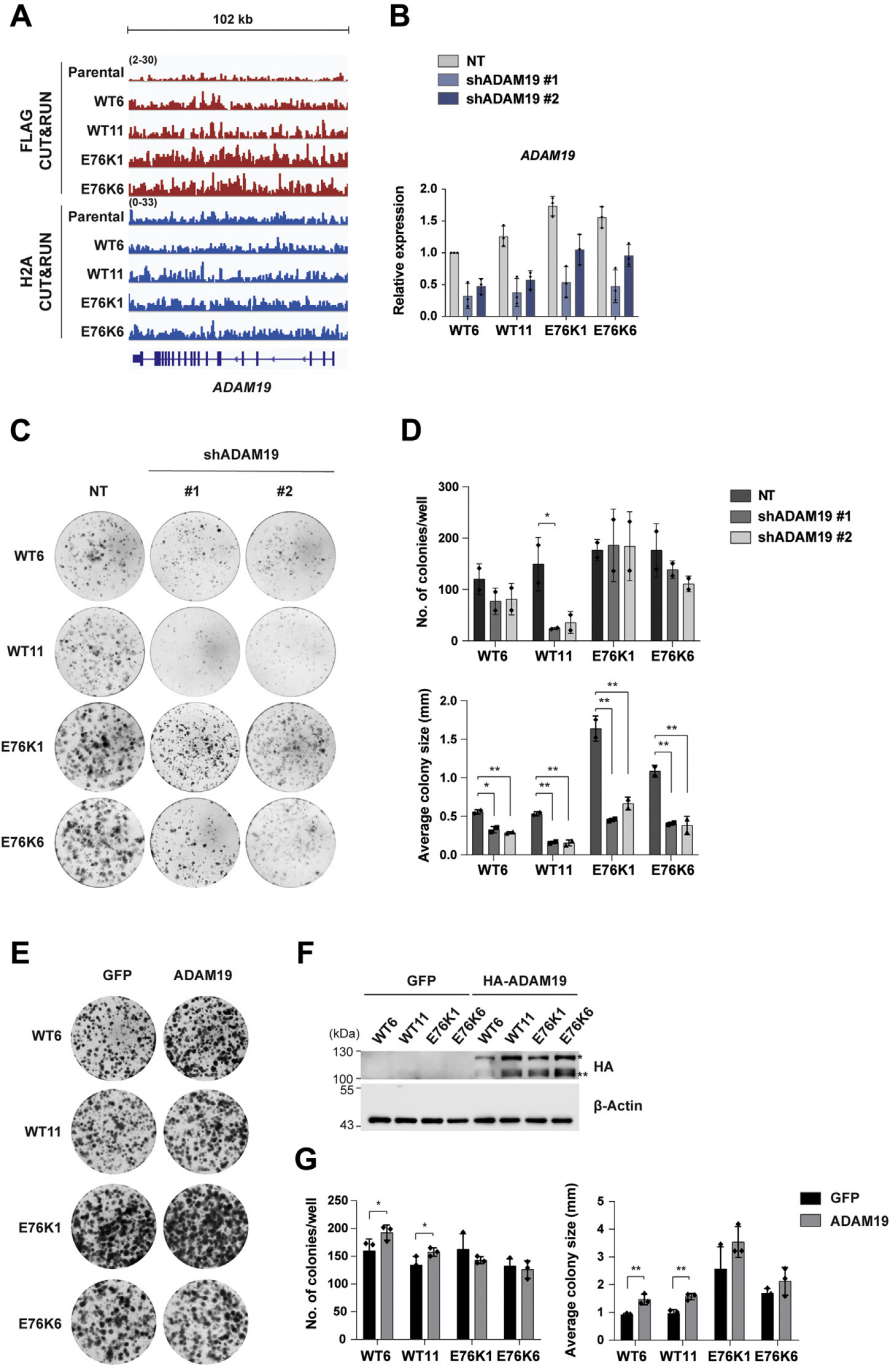
***ADAM19* upregulation promotes the colony formation ability of H2BE76K mutant cells.***A*, H2BE76K is enriched on the gene body of *ADAM19*. IGV tracks of FLAG and H2A CUT&RUN profile of *ADAM19*. *B*, mRNA expression of *ADAM19* was quantified 72 h after shRNA-mediated knockdown. Results are shown as mean ± SD from three independent experiments. *C*, knockdown of *ADAM19* decreased colony formation ability. *D*, colony number and average sizes of colonies from three replicates are shown (mean ± SD, ∗*p* ≤ 0.05, ∗∗*p* ≤ 0.01). *p*-values were calculated using one-sided *t*-test between NT and each shRNA-treated sample. *E*, ADAM19 overexpression increases sizes of WT and H2BE76K colonies. *F*, GFP and HA-tagged *ADAM19* were transiently overexpressed in the KI cells. ∗ and ∗∗ indicate the pro- and mature forms of ADAM19, respectively. β-Actin acts as loading control. *G*, colony number and average sizes of colonies from three replicates are shown (mean ± SD, ∗*p* ≤ 0.05, ∗∗*p* ≤ 0.01). *p*-values were calculated using one-sided *t*-test between samples overexpressing GFP and ADAM19.

The role of the H2BE76K mutation in cancer was further characterized by studying the oncogenic properties of the H2BE76K mutant cells. The H2BE76K mutant cell lines formed bigger colonies than the WT isogenic lines and one of the H2BE76K mutant lines had moderate increased proliferation rates (Fig. S9, *A* and *B*). RNA-seq results showed that the expression of genes that control cellular phenotypes was affected (Fig. 3*B*), which might directly contribute to the increased clonogenicity of the H2BE76K mutant cells. Indeed, several upregulated genes in the H2BE76K mutant cells, such as *WNT7B* and *PTN*, are involved in promoting metastasis-associated tumor cell phenotypes, including increased clonogenicity (24, 25) (Fig. S9*C*). To identify H2BE76K target genes that promote colony formation in the H2BE76K mutant cells, we depleted multiple H2BE76K target genes individually using lentiviral short hairpin RNAs (shRNAs). Intriguingly, we found that the knockdown of one of the H2BE76K-enriched genes, *ADAM19* (Fig. 4, *A* and *B*, Figs. S9*A* and S10), reduced the sizes of colonies formed by the H2BE76K cells to the sizes of WT colonies treated with nontargeting (NT) shRNA (Fig. 4, *C* and *D*) with minimal effect on proliferation (Fig. S9). ADAM19 belongs to the ADAM (a disintegrin and metalloproteinase) family that comprises transmembrane and secreted proteins and is found to be highly expressed in various human carcinomas including breast invasive carcinoma (BRCA) (Fig. S11). Conversely, the overexpression of ADAM19 significantly increased the sizes of colonies formed by the WT cells (Fig. 4, *E*–*G*). These results demonstrate that *ADAM19* upregulation promotes the colony formation ability of the H2BE76K cells.

### H2BE76K mutant histone allows faster Pol II progression on *ADAM19 in vivo*

**Figure 5:**
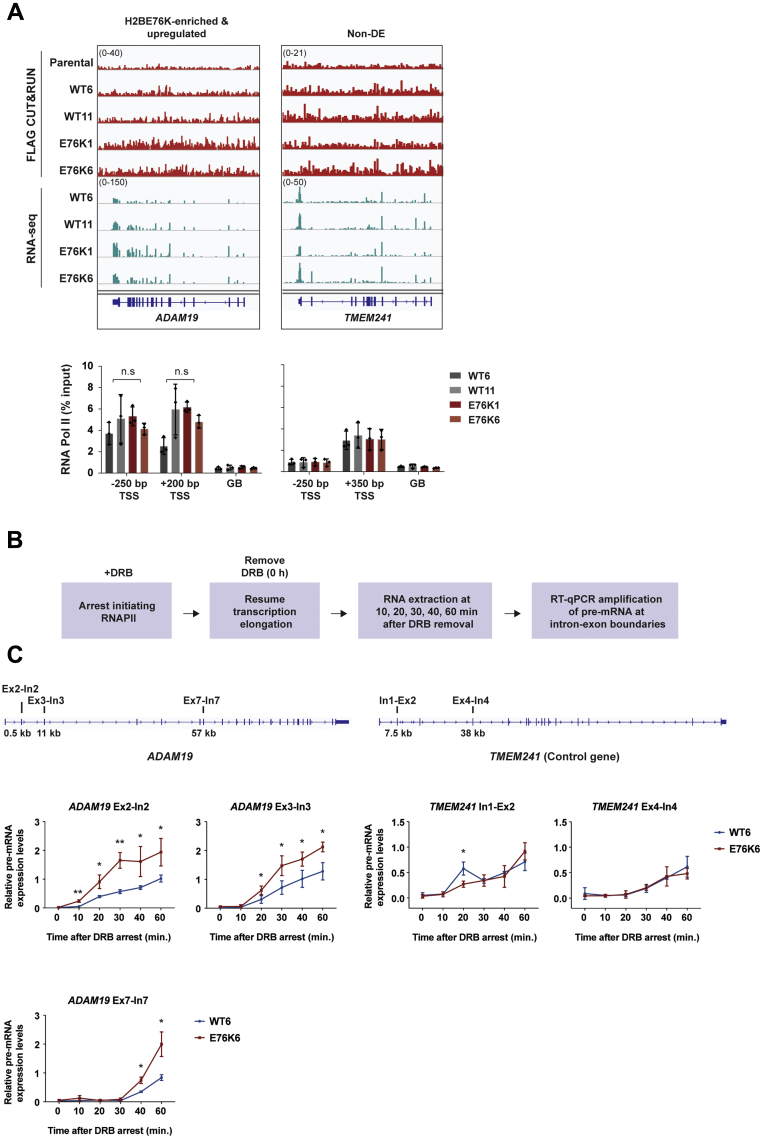
**H2BE76K directly elevates the transcription of *ADAM19 in vivo*.***A*, RNA Pol II occupancy is comparable between WT and H2BE76K mutant cells surrounding the TSS regions. Pol II ChIP-qPCR was performed to assess Pol II occupancy on the indicated regions on *ADAM19* and the negative control gene, *TMEM241*. Results from three independent experiments are shown (mean ± SD). *p*-values were calculated using one-sided *t*-test between WT and H2BE76K samples (n.s., not significant). *B*, experimental scheme of the time course DRB-qPCR to monitor transcription elongation rates. *C*, *ADAM19* shows increased Pol II transcription elongation rate. Pre-mRNA levels were measured by RT-qPCR at the indicated exon–intron (Ex-In) junctions on *ADAM19* and the control gene, *TMEM241*. Results are shown as mean ± SD from three independent experiments. *p*-values were calculated using one-sided *t*-test (∗*p* ≤ 0.05, ∗∗*p* ≤ 0.01).

We next investigated the mechanism of *ADAM19*’s upregulation in the H2BE76K mutant cells. As H2BE76K destabilizes the nucleosome, its enrichment on gene bodies may provide a more permissive environment for RNA polymerase II (Pol II) progression. As Pol II recruitment is a key step of transcription activation, we first examined Pol II occupancy on *ADAM19* by ChIP-qPCR. We monitored Pol II recruitment by treating the cells with 5,6-dichlorobenzimidazole1-β-D-ribofuranoside (DRB) to inhibit the positive transcription elongation factor-b (P-TEFb) kinase. DRB treatment inhibits initiating Pol II from entering productive elongation while allowing elongating complexes to complete transcription, effectively arresting Pol II surrounding the TSS. Pol II recruitment to *ADAM19* was comparable between WT and H2BE76K cells, suggesting an alternative mechanism driving *ADAM19*’s upregulation (Fig. 5*A*). To further examine the effect of H2BE76K on *ADAM19* transcription, we monitored *in vivo* Pol II transcription elongation by transiently inhibiting transcription elongation with DRB (26) (Fig. 5*B*). As DRB’s inhibitory effect on transcription is reversible, transcription elongation resumes upon its removal. We quantified pre-mRNA expression of *ADAM19* through RT-qPCR by using primers that span several exon–intron junctions along *ADAM19* at different time points upon DRB removal. Indeed, faster transcription elongation of *ADAM19* was observed in the H2BE76K mutant cells (Fig. 5*C*, results from the WT11/E76K1 pair are shown in Fig. S12). Pre-mRNA expression of the exon2–intron2 junction was detected after DRB removal 10 min earlier in the E76K6 cells compared with the WT6 cells. Similar phenomena were also observed in other tested intron–exon junctions, confirming the more efficient transcription elongation on *ADAM19* in H2BE76K mutant cells. In contrast, *TMEM241*, a control gene that is not targeted by H2BE76K, showed similar rates of transcription recovery after DRB removal in WT and H2BE76K cells. Taken together, these results revealed the direct role of H2BE76K in enhancing the transcription of *ADAM19*, which contributed to the increased oncogenic properties in H2BE76K mutant cells.

## Discussion

Our results show that the H2BE76K mutation exerts significant effects on the transcriptome and cellular phenotypes of breast cancer cells. Other well-known oncohistone mutations are located on or adjacent to sites that are posttranslationally modified, thereby mediating their effect through PTM perturbations. In contrast, the E76 residue on H2B is not known to be posttranslationally modified (18), and overall levels of common histone modifications were not affected in the H2BE76K mutant breast cancer cell lines used in this study. Our data demonstrate the mechanism by which a cancer-associated mutation promotes oncogenic properties by directly altering the transcriptome through a combination of its nucleosome destabilizing effect and preferential localization.

Our immunoprecipitation experiments complemented the results of crystallography analyses and *in vitro* studies that revealed the disruptive effect of the H2BE76K mutation on H2B-H4 interaction (16, 17). Despite the local nucleosome destabilization effect mediated by H2BE76K, both nucleosomal occupancy and chromatin accessibility appeared unaltered in H2BE76K-enriched regions. We reasoned that the H2BE76K-containing nucleosomes remain relatively stable *in vivo* and that the local nucleosome destabilization effect might not necessarily translate to detectable chromatin accessibility changes in the H2BE76K mutant cells used in this study. Although a small portion of the H2BE76K failed to deposit on the chromatin, most H2BE76K mutant protein was incorporated into the chromatin (Fig. 1*G*, Fig. S4). In addition, genomic mapping by CUT&RUN revealed that the H2BE76K mutant histone is redistributed preferentially to genic regions. As of now, the mechanism of H2BE76K deposition remains unclear.

During transcription elongation, the nucleosome is partly disassembled through the removal of one H2A-H2B dimer to facilitate Pol II progression (27, 28). As H2A/H2BE76K dimers are likely to be more easily displaced during Pol II progression, the presence of H2BE76K on gene body regions may help to relieve the nucleosomal barrier, resulting in a more transcriptionally permissive environment. In line with this observation, our findings revealed a positive correlation between H2BE76K enrichment and elevated gene expression. It is important to note that the FLAG-tagged H2BE76K is expressed by one out of 14 somatic H2B encoding genes expressed in our KI cell lines and only constitutes a fraction of total H2B. Total H2B, as reflected by H2A occupancy, remained unchanged in H2BE76K-enriched regions. The increased H2BE76K enrichment signifies a higher H2B fraction within the H2B population in any given genomic region and could likely therefore exert a stronger effect on transcription.

A closer examination on the relationship between H2BE76K and transcription showed that Pol II progressed more efficiently on H2BE76K-enriched *ADAM19*. Therefore, we propose a model by which the H2BE76K mutant histone preferentially localizes to *ADAM19*, subsequently promoting transcription by facilitating H2A-H2B dimers displacement/exchange during Pol II progression. Furthermore, depletion of the repressive mark H2AK119ub and enrichment of the active mark H3K27ac in H2BE76K containing nucleosomes provided additional evidence to a positive association between H2BE76K and transcription.

In agreement with previous studies (16, 17), our data show that the cellular phenotypic changes brought on by H2BE76K enhance colony formation ability of breast cancer cells. Indeed, many genes that control cellular properties and signaling were differentially expressed in the H2BE76K mutant cells. The increased expression of *ADAM19* was identified as one of the contributing factors. We found that the H2BE76K-enriched gene *ADAM19* is upregulated and contributed to the enhanced colony formation ability of the H2BE76K mutant cells, an effect that was reversed upon *ADAM19* knockdown. ADAM proteins are proteolytically active and modulate cell–cell signaling through proteolytic cleavage of growth factors and ligands (29). In mice, *Adam19* knockout resulted in defects in heart development, due in part to the impaired epithelial–mesenchymal transition (EMT) of endocardial epithelial cells (30, 31). The biological function of *ADAM19* suggests that it might play an important role in mediating cell–cell signaling and that changes in its expression would alter cell–cell interaction and movement (32). Indeed, high *ADAM19* expression was shown to be associated with brain metastasis in breast cancer (33), suggesting that the increased *ADAM19* expression promotes metastatic potential of the H2BE76K mutant cells. Nonetheless, we do not rule out the significance of the effects of other dysregulated genes in H2BE76K mutant breast cancer cells. The nearly 500 downregulated genes found in H2BE76K mutant cells might also contribute to oncogenic properties and subsequently cancer development. We found three H2BE76K-enriched and upregulated genes *HIPK2*, *PTCH1*, *TRPS1* that are associated with GO term “negative regulation of transcription by RNA polymerase II” using the DAVID functional annotation clustering function. TRPS1 is a known GATA transcriptional repressor that is implicated in breast cancer (34, 35). RNA-seq analysis of the HCC3153 and SUM159 triple-negative breast cancer cell lines identified 925 and 664 common up- and downregulated genes (adjusted *p* < 0.05) after *TRPS1* knockdown (35). By comparing the differentially expressed genes, we found that 12% of the H2BE76K up- (228 genes) and downregulated (493 genes) genes overlapped with genes differentially expressed after TRPS1 knockdown (Fig. S13). This broadly indicates that *TRPS1* upregulation might play a role in gene repression and activation in our H2BE76K cells. Moreover, some of the downregulated genes found in the H2BE76K mutant cells such as *JUP* (36, 37) and *TRIM29* (38, 39) act as tumor suppressors in breast cancer, suggesting that the repressed genes might also contribute to oncogenic properties.

Our data highlight the role of a nucleosome destabilizing histone mutation and its subsequent contribution to breast cancer progression, as well as present the H2BE76K mutation as an interesting model to study the *in vivo* effects of nucleosome instability on transcription. In this study, we provide the genome-wide localization pattern of the H2BE76K mutant histone, showing its preferential deposition in genic regions. The increased enrichment of H2BE76K in gene bodies directly elevates the transcription of genes that promote colony formation ability of breast cancer cells. We speculate that similar mechanisms underlie other carcinomas containing the H2BE76K mutation, as well as other nucleosome destabilizing histone mutations, particularly the H2BE76 interacting H4R92 and H4D68 residues that were also found to be mutated in various cancers (15).

## Experimental procedures

### Plasmid construction

Genomic regions downstream of the start codon and downstream of the stop codon were scanned for suitable gRNA target sequences with minimal off-target binding sites using http://crispr.mit.edu/. The 5’ and 3’ sgRNA guide sequences were cloned into lentiCRISPRv2 as described (40). The sgRNA sequences are shown in Table S2. The pBlueScript donor template includes the 5’ and 3’ homology arms, FLAG-*HISTH2BO*, IRES, and Neomycin resistance gene sequences, which were cloned using restriction sites. The 1 kb 5’ and 3’ homology arms correspond to the regions flanking the 5’ and 3’ PAM sites. The FLAG-H2BO carries either the WT (E76) or the H2BE76K (K76) mutation. Human ADAM19 ORF was cloned into a pLenti-CMV-puro backbone for overexpression in MDA-MB-231 KI cells.

### Cell culture

293T and MDA-MB-231 cells were maintained in Dulbecco’s modified Eagle’s medium (DMEM) supplemented with 10% FBS. Cells were cultured at 37 °C with 5% CO_2._

### Generation of CRISPR/Cas9 knock-in cell lines

The pBlueScript donor construct and two lentiCRISPRv2 constructs carrying 5’ and 3’ sgRNA each were transfected into MDA-MB-231 using Lipofectamine 2000. The cells were split 1:5 24 h after transfection and subjected to G418 (2 mg/ml) selection 24 h later. Single clones were picked under the microscope.

### CRISPR/Cas9 clones genotyping

Positive clones were confirmed through PCR genotyping. Genomic DNA was extracted from clones with the MiniBEST Universal Genomic DNA Extraction Kit (TaKaRa). KI clones were screened for successful 5’ and 3’ integration at the respective PAM sites, as well as successful integration of exogenous sequence using PCR. The integration and insertion sites were amplified by PCR with three sets of primers listed in Table S3. PCR products were purified and sequenced to confirm correct sequences and successful integration at the target location.

### CRISPR/Cas9 off-target analysis

The top five potential off-target sites of each sgRNA predicted by http://crispr.mit.edu/ were analyzed. The sites were amplified by PCR and sequenced.

### Colony formation assay

Each cell line was seeded in triplicates wells at a density of 800 cells/well in 6-well plates. Cells treated with shRNA or overexpressing GFP/ADAM19 were kept under puromycin selection. After 12 days, the cells were fixed with ice cold methanol for 10 min and stained with 0.5% crystal violet for 15 min. Number of colonies per well and colony size were quantified using ImageJ.

### Proliferation assay

In total, 3000 cells were seeded in triplicates in a 96-well plate. Medium containing 10% CCK-8 solution was incubated with the cells for 2.5 h before measurement at OD450. Measurements were taken 24, 48, 72 h after cell seeding.

### shRNA transfection

293T cells were transfected using Polyethylenimine (PEI) with pLKO.1 shRNA constructs and packaging viruses, pVSVG and pEXQV. Viruses were collected 48 h after transfection and used to infect MDA-MB-231 cells. Infected cells were maintained in 1 μg/ml puromycin supplemented medium for 72 h before RNA extraction. shRNA sequences used for the study are listed in Table S4.

### GFP and ADAM19 overexpression

293T cells were transfected using PEI with pLenti-CMV-puro constructs carrying GFP and *ADAM19* and packaging viruses, pVSVG and pEXQV. Viruses were collected 48 h after transfection and used to infect MDA-MB-231 cells. After 24 h, MDA-MB-231 cells were reseeded for colony formation assays.

### Chromatin fractionation

Cells were trypsinized, collected, and washed twice with cold PBS. The cell pellet was lysed in Buffer A (10 mM HEPES, pH7.9, 10 mM KCl, 1.5 mM MgCl_2_, 0.34 M Sucrose, 10% Glycerol, 1 mM DTT, Protease Inhibitor Cocktail) containing 10% Triton X-100 on ice for 8 min and centrifuged for 5 min at 1300*g*. The cytosolic supernatant was cleared for 5 min at 20,000*g* (S2 fraction). The nuclear pellet was washed with Buffer A three times before lysis in Buffer B (3 mM EDTA, 0.2 mM EGTA, 1 mM DTT, Protease Inhibitor Cocktail) for 30 min on ice. Nuclear lysate was centrifuged for 5 min at 1700*g* to separate insoluble and soluble chromatin fractions (S3 fraction). The insoluble chromatin fraction was washed three times with Buffer B, resuspended in cold PBS, and sonicated (P3 fraction). The samples were analyzed by immunoblotting.

### 293T FLAG-IP

293T cells were transfected with pcDNA3.1(+) constructs expressing *HIST1H2BO* (WT, E76A, E76K, E76Q). Cells were collected 48 h post transfection. The cells were washed three times with cold PBS before lysing for 30 min in lysis buffer (20 mM Tris pH 8,0, 150 mM NaCl, 1 mM EDTA, 1% Triton X-100). The lysate was sonicated with a Branson sonifier (20% Amplitude, 3 × 10 s pulse, 1 min rest between pulses). Cleared lysate was incubated overnight with Anti-FLAG M2 Beads (Sigma), followed by three washes in lysis buffer. Sample buffer was added to the beads and boiled for 5 min at 95 °C. The samples were analyzed by immunoblotting.

### Mononucleosome IP (fixed cells)

293T cells were fixed with 1% PFA for 5 min and then quenched with 125 mM glycine for 5 min at RT. Cells were lysed in extraction buffer (10 mM Tris pH 7.5, 10 mM NaCl, 0.5% NP-40, Protease Inhibitor Cocktail) on ice for 45 min. Chromatin was digested with MNase in digestion buffer (20 mM Tris HCl pH 7.5, 15 mM NaCl, 60 mM KCl, 2 mM CaCl_2_) to obtain mononucleosomal sized DNA. Mononucleosomes were immunoprecipitated with anti-FLAG M2 beads in IP buffer (50 mM Tris pH 8, 20 mM EDTA, 100 mM NaCl, 1% Triton X-100) overnight. Beads were washed 3× with IP buffer. Proteins were eluted by boiling the beads in sample buffer and analyzed by immunoblotting.

### Mononucleosome IP (native chromatin)

MDA-MB-231 cells were collected and washed twice with cold PBS. The cells were lysed in hypotonic buffer (10 mM HEPES pH7.4, 10 mM KCl, 0.05% NP40, Protease Inhibitor Cocktail) for 20 min on ice. Cytosolic and nuclei fractions were separated by centrifugation at 300*g* for 10 min. Nuclei were washed once (20 mM HEPES, 20 mM KCl, 0.5 mM EDTA) and washed again (20 mM HEPES pH 7.4, 20 mM KCl, 0.5 mM EDTA, 300 mM NaCL) before resuspension in MNase digestion buffer (20 mM HEPES pH 7.4, 20 mM KCl, 0.5 mM EDTA, 300 mM NaCl, 3 mM CaCl_2_). The chromatin was digested with MNase at 37 °C for 90 min to mononucleosomes before adding 5 mM EGTA and 0.05% NP40 to stop the MNase reaction. Both cytosolic and mononucleosome fractions were centrifuged for 30 min at 20,000*g*. Cytosolic fraction was supplemented with 150 mM NaCl. Cleared lysates were incubated with anti-FLAG M2 beads overnight. The FLAG beads were washed once with Buffer A (20 mM HEPES pH 7.4, 20 mM KCl, 0.4 mM EDTA), Buffer A (supplemented with 300 mM KCl), and lastly with Buffer A (supplemented with 300 mM KCl and 0.1% Tween). Sample buffer was added to the beads and boiled for 5 min at 95 °C. Protein samples were analyzed by immunoblotting.

### Acid histone extraction

Cells were washed with cold PBS supplemented with 5 mM sodium butyrate and lysed in extraction buffer (PBS, 0.5% Triton X-100, 2 mM PMSF, 0.02% NaN3) for 10 min. Chromatin pellets were dissolved in 0.2 N HCl overnight at 4 °C. Supernatant was collected after centrifugation and neutralized with 2N NaOH.

### Immunoblotting

Whole-cell lysates or protein samples were separated by SDS-PAGE and transferred onto nitrocellulose membrane. The membranes were blocked with 5% nonfat milk/TBST and incubated with primary antibodies overnight followed by incubation with HRP-conjugated secondary antibodies. Proteins were detected by enhanced chemiluminescence. Protein levels were quantified using ImageJ.

### CUT&RUN

CUT&RUN was performed according to the protocol using the high-calcium/low-salt digestion conditions described in Skene and Henikoff (2017) (21) and Meers *et al.* (2019) (41) with slight modifications. All reactions were performed at 4 °C unless stated otherwise. Each centrifugation step was performed at 600*g* for 3 min. In total, 4 × 10^6^ cells were used for each reaction. Cells were washed twice with PBS and resuspended in Buffer NE1 (20 mM HEPES-KOH pH 7.9, 10 mM KCl, 0.5 mM Spermidine, 0.1% Triton X-100, 20% Glycerol, Proteinase Inhibitor Cocktail). After 10 min, cells were collected and incubated in Buffer 1 (20 mM HEPES-KOH pH 7.9, 150 mM NaCl, 2 mM EDTA, 0.5 mM Spermidine, 0.1% BSA, Proteinase Inhibitor Cocktail) for 5 min. Cells were collected, washed once with Buffer 2 (20 mM HEPES pH 7.5, 150 mM NaCl, 0.5 mM Spermidine, 0.1% BSA, Proteinase Inhibitor Cocktail), and resuspended in Buffer 2. Samples were incubated with antibodies for 2 h or overnight. Samples containing primary mouse antibodies were washed twice with Buffer 2 before incubation with anti-mouse rb IgG for 1 h. Nuclei was collected, washed twice with Buffer 2, and incubated with pA-MNase fusion protein (150 ng) for 1 h. Nuclei was washed once with Buffer 2 and once with low-salt buffer (3.5 mM HEPES, Proteinase Inhibitor Cocktail). Upon resuspension in low-salt buffer, 10 mM CaCl_2_ was added to each reaction. MNase digestion was stopped after 30 min with the addition of Buffer 2 (supplemented with 10 mM EDTA, 20 mM EGTA and 28.8 pg yeast spike in). Samples were incubated for 15 min at 37 °C. DNA was purified using NucleoSpin Gel and PCR Clean-up kit (MACHEREY-NAGEL). CUT&RUN libraries were prepared using Ovation Ultralow System V2 (NuGEN) followed by quality control using Bioanalyzer 2100. Libraries were sequenced on a HiSeq platform (Illumina) using paired-end sequencing.

### ATAC-seq

ATAC-seq was performed according to the protocol in Buenrostro *et al.* (42). In total, 50,000 cells were suspended in cold lysis buffer (10 mM Tris-HCl pH 7.4, 10 mM NaCl, 3 mM MgCl_2_, 0.1% NP40). Crude nuclei were isolated by centrifugation at 500*g* for 10 min and resuspended in 25 μl of TD Buffer (2× reaction buffer, Illumina). Nuclei pellet was collected (500*g*, 5 min) and suspended in transposition reaction mix (25 μl, 2.5 μl TDE1, 22.5 μl H_2_O). The reactions were incubated at 37 °C for 30 min with gentle mixing. DNA was immediately purified using Qiagen MinElute PCR Purification kit. Libraries were PCR amplified according to protocol and purified using Qiagen MinElute PCR Purification kit. Double-sided bead purification (0.5× beads volume, followed by 1.3× beads volume) was performed using AMPure XP beads to enrich for fragments between 150 bp and 1000 bp. Library quality and fragment sizes were accessed using Bioanalyzer (Agilent). ATAC-seq libraries were sequenced with 150 bp paired-end sequencing on a HiSeq platform.

### ChIP-qPCR

For FLAG and H3 ChIP, 231 KI cell lines were fixed with 1% PFA for 5 min at RT and then quenched with 125 mM glycine for 5 min. Cells were lysed in extraction buffer (10 mM Tris pH 7.5, 10 mM NaCl, 0.5% NP-40, Protease Inhibitor Cocktail) on ice for 45 min. Chromatin was subjected to light MNase digestion in digestion buffer (20 mM Tris HCl pH 7.5, 15 mM NaCl, 60 mM KCl, 2 mM CaCl_2_) to obtain equal populations of mono-, di-, and tri-nucleosomal sized DNA. Chromatin was immunoprecipitated with αFLAG M2 beads and αH3 antibody in ChIP buffer (50 mM Tris pH 8, 20 mM EDTA, 100 mM NaCl, 1% Triton X-100, 0.1% sodium deoxycholate) overnight. Chromatin bound to antibody was captured with protein G beads for 2 h and washed (1× ChIP buffer, 1× ChIP buffer +500 mM NaCl, 1× Tris/LiCl buffer, 2× Tris/EDTA buffer).

For DRB-RNA Pol II ChIP, 231 KI cell lines were treated with 150 μM DRB (5,6-dichloro-1-beta-D-ribofuranosylbenzimidazole) for 3 h. Cells were fixed with 1% PFA for 5 min at RT, quenched with 125 mM glycine for 5 min, and lysed in ChIP buffer (50 mM HEPES pH 7.5, 1 mM EDTA, 140 mM NaCl, 1% Triton X-100, 0.1% sodium deoxycholate). Chromatin was sonicated with Diagenode Bioruptor for 30 cycles (30 s on/off at high power setting) to 100 to 300 bp sized DNA. Cell lysates were immunoprecipitated with IgG or anti-Rbp1 antibody overnight and subsequently incubated with protein G beads for 2 h. Beads were washed once with ChIP buffer, once with ChIP buffer (+500 mM NaCl), once with Tris/LiCl buffer, and twice with Tris/EDTA buffer.

ChIP DNA was extracted using the Chelex method (43) and eluted in 10 mM Tris pH 8. Input and ChIP DNA were quantified using qPCR. ChIP enrichment was calculated by normalizing the values of ChIP samples relative to that of the input samples using the 2^−ΔCt^ method. Primers used are listed in Table S6.

### RNA extraction, RNA-Seq library preparation

Total RNA was extracted from cells using the MiniBEST Universal RNA extraction kit (Takara). Ribosomal RNA was depleted removed using the NEBNext rRNA Depletion Kit (NEB). The libraries were prepared with NEBNext Ultra II Directional RNA Library Prep Kit for Illumina (NEB) according to the manufacturer’s protocol, followed by quality control using Bioanalyzer 2100. The libraries were sequenced on a HiSeq platform (Illumina) using 150 bp paired-end sequencing on a HiSeq platform.

### Quantitative reverse transcription PCR (RT-qPCR)

cDNA was reverse transcribed from total RNA using the PrimeSript RT Master Mix (Takara). qPCR was performed on Applied Biosystems QuantStudio 3 Real-Time PCR Systems. The qPCR reactions were set up with SYBR Green (Takara) in technical duplicates. Relative fold expression was calculated using the 2^−ΔΔCt^ method with *ACTIN* as the endogenous control. Primers used for RT-qPCR are listed in Table S5.

### DRB-qPCR

Cells were treated with 150 μM DRB for 3 h. Medium containing DRB was removed and cells were washed twice with PBS before replacing with fresh medium. Total RNA was extracted at indicated time points after DRB removal. RNA was reverse transcribed to cDNA and quantified with qPCR. Pre-mRNA levels were calculated using the 2^−ΔΔCt^ method with *ACTIN* mRNA as the endogenous control and normalized relative to the value of the pretreatment WT samples, which is set as 1. Primers used are listed in Table S7.

### Antibodies

Antibodies used for CUT&RUN are αH2A (Abcam, 15653), αFLAG (Sigma, F1804), αmouse rb IgG (Jackson ImmunoResearch, 315-005-003). Primary antibodies used for immunoblotting include: αγH2Ax (Milipore, ab26350), αFLAG (Sigma, F7425), αH2B (CST, D2H6), αH4 (CST, D2X4V), αH3 (Immunoway, YM3038), αNAP1L1 (Abcam, ab33076), αNAP1L2 (Abnova, H00004674-D01), αSPT16 (Santa Cruz, sc-377028), αSSRP1 (Santa Cruz, sc-74536), αHSC70 (Santa Cruz, sc-7298), αC23 (Santa Cruz, sc-55486), αH2BK120ub (CST, D11), αH2AK119ub (CST, D11), αH3K4me3 (Active Motif, 61379), αH3K36me3 (Immunoway), αH3K36me2 (CST, 29015), αH3K9me3 (Abcam, ab8898), αH3K27me3 (CST, 9733), αH4K16ac (Immunoway, YK0014), αH4K20me3 (Active Motif, 39671), αH3K27ac (Active Motif, 39685), αH3K79me3 (Immunoway, YM3091), αH3K9ac (Immunoway, YK0006), αHA (Santa Cruz, SC7392), αβ-Actin (Immunoway, YM3028). αFLAG M2 Affinity Gel (Sigma), αH2A (Abcam, 15653), and αRpb1 (CST, 14958) were used for ChIP.

### CUT&RUN data analysis

CUT&RUN data were analyzed using peak calling analysis, differential gene enrichment analysis, and genome-wide differential enrichment analysis. Adapters were removed by “cutadapt” (44). Reads that passed the quality filter were then aligned to human reference genome *hg19* and *Saccharomyces cerevisiae* reference genome *sacCer3* by “Bowtie2” (v2.3.4.1) separately with parameters *“--local --very-sensitive-local --no-unal --no-mixed --no-discordant --phred33 -I 10 -X 700”* (45). The normalization factor used in this paper was calculated as 1 over the number of reads mapping to *Saccharomyces cerevisiae* genome per 100,000 (Spike-in normalized). Spike-in normalized coverage data was then generated by “deepTools” (v3.2.1) based on the normalization factors (46). “MACS2” was used to call peaks using parental sample as control under *q* < 0.05 with pair-end and broad peaks mode (47). Peak annotation to different genomic regions, including gene body, promoter (TSS ± 3kb), and distal intergenic regions, was performed by R package “ChIPseeker” (48). *Chi-square* test was then performed to assess the statistical significance in the difference of genomic distributions between H2BE76K and WT samples. For each gene, the occupancy of FLAG was quantified by the number of reads located in gene body using “BEDTools” (49) and subsequently normalized by the spike-in normalization factor. To identify genes showing differential occupancy of FLAG between H2BE76K and WT samples, differential occupancy analysis was further performed by “DESeq2” (Benjamini-Hochberg (BH) adjusted *p* < 0.05, log_2_ fold enrichment >0.5) (50).

To investigate the genome-wide differential FLAG-H2B enrichment, we calculated the read counts for FLAG within each bin with 10 kb using the function “multiBamSummary bins” in deeptools (46). Differential enrichment of FLAG-H2B at each bin was performed by DESeq2 (50). Threshold of log2 fold change ±0.58 (1.5-fold change) was used to define gain or loss bins between H2BE76K mutant samples and WT samples. To avoid overestimating, we ignored bins with counts lower than the median value in each sample. Bins were annotated as promoter, genic, or intergenic based on the overlaps with promoter (±1.25kb of the TSS), genic (overlapped with gene region), and intergenic regions (the complement of promoter and genic regions) using the gene annotation for Ensembl’s reference (GRCh37; n = 62,475 genes). The significance of difference for bin genome-wide distributions between gain and loss bins was evaluated by the Chi-square test.

### RNA-seq data analysis

Adapters were first removed by “cutadapt” (44). Reads that passed the quality filter were then aligned to human reference genome *hg19* from UCSC and counted by “STAR” (v2.6.1a) with default parameters (51). Differentially expressed genes between H2BE76K samples and WT samples were identified by R package “DESeq2” (BH adjusted *p* < 0.05, absolute log_2_FC > 0.25) (50). Gene ontology analysis based on upregulated genes or downregulated genes was performed by R package “HTSanalyzeR2” (52). Read counts were normalized using the variance stabilized transformation (VST) method in “DESeq2” (50). The VST-transformed gene expression levels were clustered using Euclidean distance and hierarchical clustering with the complete linkage method to produce heatmap.

### ATAC-seq data analysis

Adapters were first removed by “cutadapt” (44). Reads that passed the quality filter were then aligned to human reference genome *hg19* from UCSC by “BWA” with “MEM” mode (53). Properly mapped reads were further used to remove duplications and reads mapped to mitochondrial chromosome. ATAC-seq reads were separated based on fragment lengths: reads longer than 100 bp are considered nucleosomal signals, and reads below 100 bp are considered nucleosome-free. Reads were further normalized using Bins per Million mapped reads (BPM) by “deepTools” (v3.2.1) (46).

### *ADAM19* gene expression analysis in TCGA

Gene expression data annotated by human reference genome *hg38* of 33 cancers were downloaded from TCGA by R package “TCGAbiolinks” (54). Data was first annotated and log_2_ transformed. Two-sided *t*-test was used to evaluate the statistical significance of *ADAM19* gene expression difference between tumor samples and normal samples within each cancer type.

## Data availability

RNA-seq, CUT&RUN, ATAC-seq data have been deposited to the NCBI Gene Expression Omnibus (GEO) in the SuperSeries under accession number GSE132749. The rest of the data is presented within the article.

## Supporting information

This article contains supporting information (21, 42).

## Conflict of interest

The authors declare that they have no conflicts of interest with the contents of this article.
