# Supplementary material for: The elevated transcription of ADAM19 by the oncohistone H2BE76K contributes to oncogenic properties in breast cancer
Source: J Biol Chem. 2021 Feb 4;296:100374. doi: 10.1016/j.jbc.2021.100374 (PMC7949156; doi:10.1016/j.jbc.2021.100374)
Supplement: Supplemental Figures S1–S13 and Tables S1–S9 [file mmc1.docx]

*Supporting information*

The elevated transcription of ADAM19 by the oncohistone H2BE76K contributes to oncogenic properties in breast cancer

Tze Zhen Evangeline Kang^1,2^*, Lina Zhu^1,2^*, Du Yang^3^, Dongbo Ding^4^, Xiaoxuan Zhu^1,2^, Yi Ching Esther Wan^1,2,^ Jiaxian Liu^1,2^, Saravanan Ramakrishnan^1,2^, Landon Long Chan^5^, Siu Yuen Chan^6^, XinWang^1,2^, Haiyun Gan^7^, Junhong Han^8^, Toyotaka Ishibashi^4^, Qing Li^3^, Kui Ming Chan^1,2#^.

**^1^** Department of Biomedical Sciences, City University of Hong Kong, Hong Kong, China

**^2^** Key Laboratory of Biochip Technology, Biotech and Health Centre, Shenzhen Research Institute of City University of Hong Kong, Shenzhen, China

^3^State Key Laboratory of Protein and Plant Gene Research, School of Life Sciences and Peking-Tsinghua Center for Life Sciences, Peking University, Peking, China

^4^Division of Life Science, Hong Kong University of Science and Technology, Hong Kong, China

^5^Department of Oncology, Princess Margaret Hospital, Hong Kong, China

^6^Department of Paediatrics and Adolescent Medicine, The University of Hong Kong, Hong Kong, China

^7^Guangdong Provincial Key Laboratory of Synthetic Genomics, CAS Key Laboratory of Quantitative Engineering Biology and Shenzhen Key Laboratory of Synthetic Genomics, Shenzhen Institute of Synthetic Biology, Shenzhen Institutes of Advanced Technology, Chinese Academy of Sciences, Shenzhen, China

^8^State Key Laboratory of Biotherapy and Cancer Center, West China Hospital, West China Medical School, Sichuan University, Sichuan, China

**#**Corresponding author: Kui Ming CHAN

[Ming.chan@cityu.edu.hk](mailto:Ming.chan@cityu.edu.hk) (K.M.C.)

Phone: 852-34424346

*These authors contributed equally to this work

# Supporting information:

Figure S1-13

Table S1-9

**Figure S1**


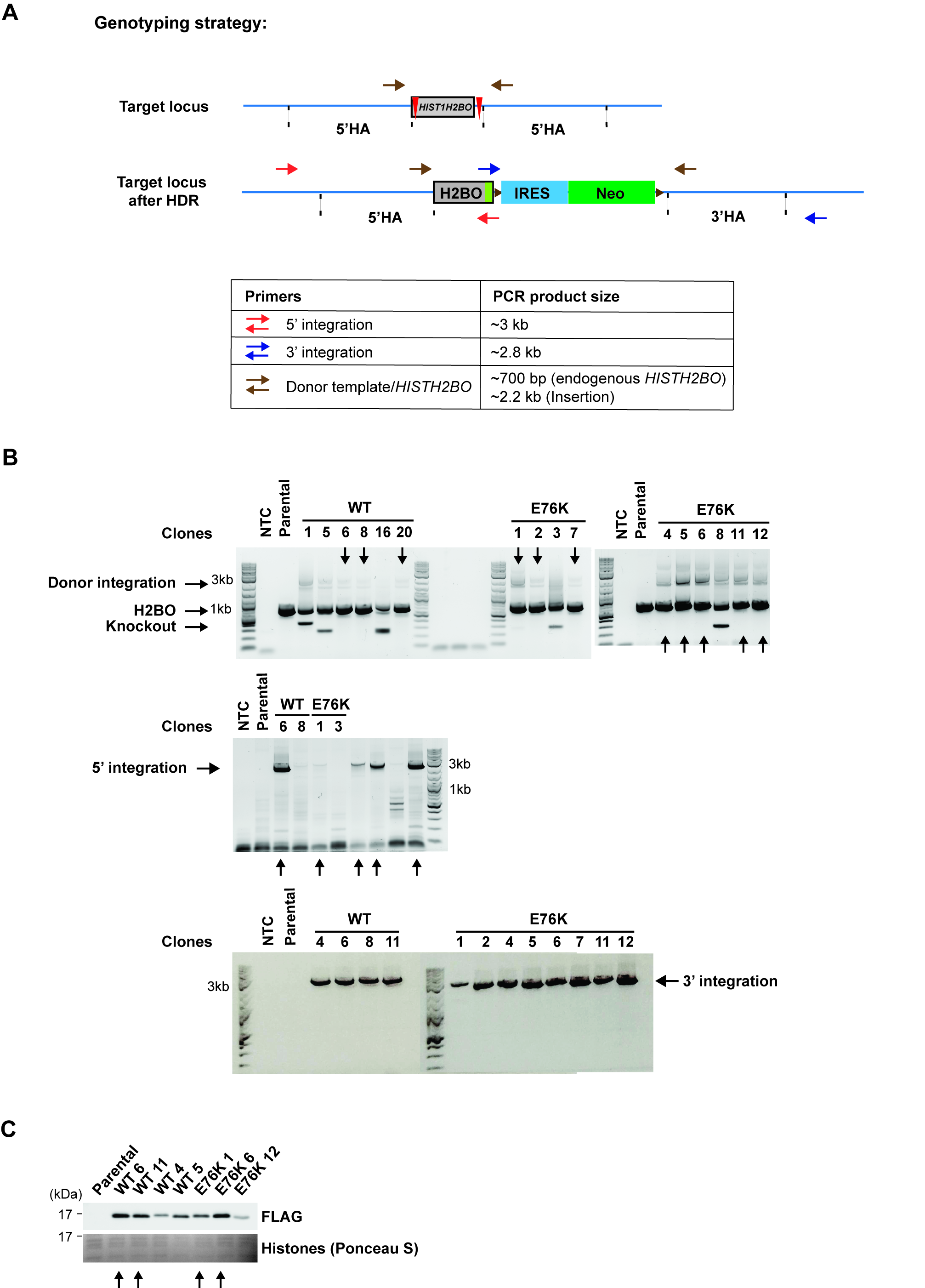


**Generation of CRISPR/Cas9 knock-in (KI) cell lines.** (**a)** Scheme of PCR-based screening of potential mutants. The target locus after CRISPR/Cas9 mediated HDR. The 5’ and 3’ HA of the donor DNA is depicted in relation to the genomic region. The arrows show the primers used for PCR genotyping to screen for positive clones. Three sets of primers were used to confirm 5’ and 3’ integration at the PAM sites, and the *HIST1H2BO* to show insertion/deletion/heterozygosity. The expected sizes of the PCR products are shown. **(b)** Genomic DNA was extracted from each clone and subjected to PCR amplification. Agarose gel picture showing PCR products amplified using a primer pair flanking the insertion site (top panel). PCR products of different sizes correspond to endogenous *HIST1H2BO*, the *HIST1H2BO*-IRES-Neo insert, and truncated *HIST1H2BO* (through deletion). PCR amplification with another two sets of primers confirmed 5’ and 3’ integration at the PAM sites (bottom panels). Arrows indicate clones with the correct genotype. **(c)** Immunoblot analysis of FLAG-H2B expression in the KI cell lines. KI cell lines expressing comparable protein levels of FLAG-H2B were selected for subsequent experiments. Arrows indicate the selected KI lines used.

# Figure S2

#
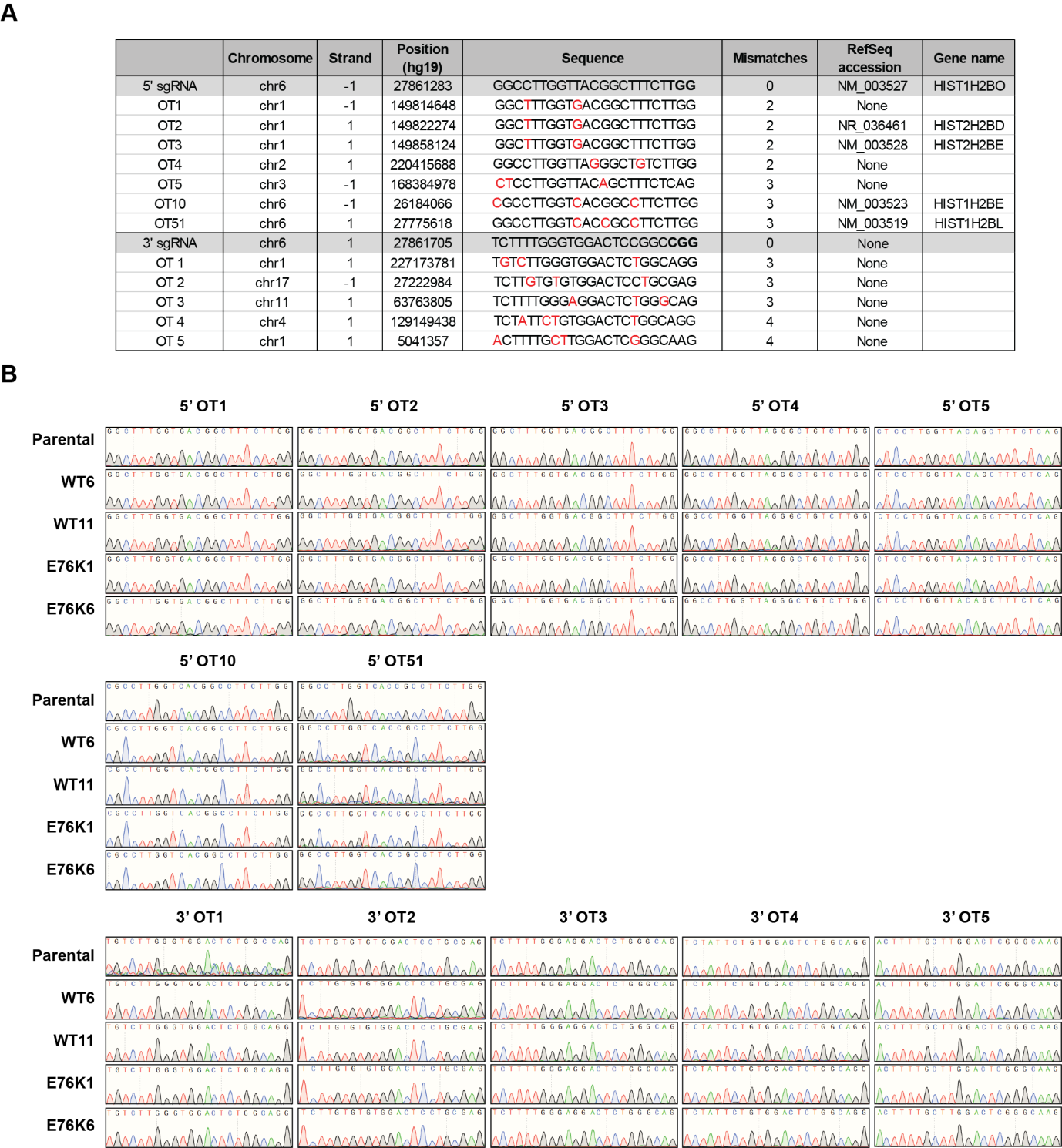


**Knock-in cell lines show minimal CRISPR-Cas9 off-target effects**. **(A)** Top 5 predicted CRISPR/Cas9 off target (OT) sites and OT sites including H2B encoding genes shown with the indicated (red) mismatches to the respective 5’ and 3’ sgRNAs. **(B)** Predicted off target sites were examined by PCR amplification using primer pairs surrounding the sites followed by sanger sequencing. Sanger sequencing chromatograms show these sites in the parental MDA-MB-231 and KI cell lines. All tested sites were unaffected in all KI lines used for this study.

# Figure S3


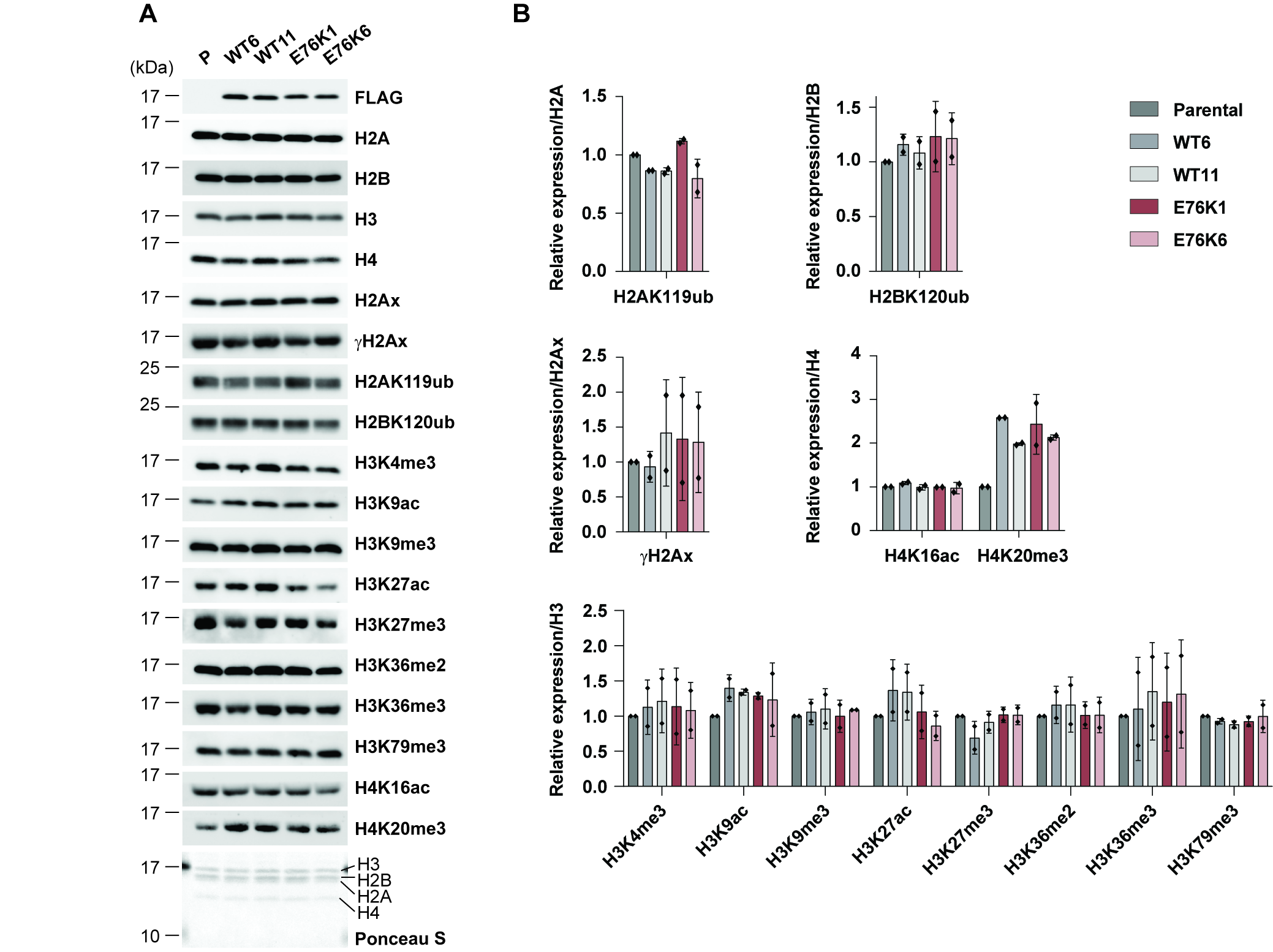


**H2BE76K does not affect global histone modification levels**. **(A)** Immunoblots of acid-extracted histones from parental MDA-MB-231 and KI cell lines. **(B)** Histone modifications were quantified relative to H2A, H2Ax, H2B, H3 or H4 levels from two independent experiments.

# Figure S4


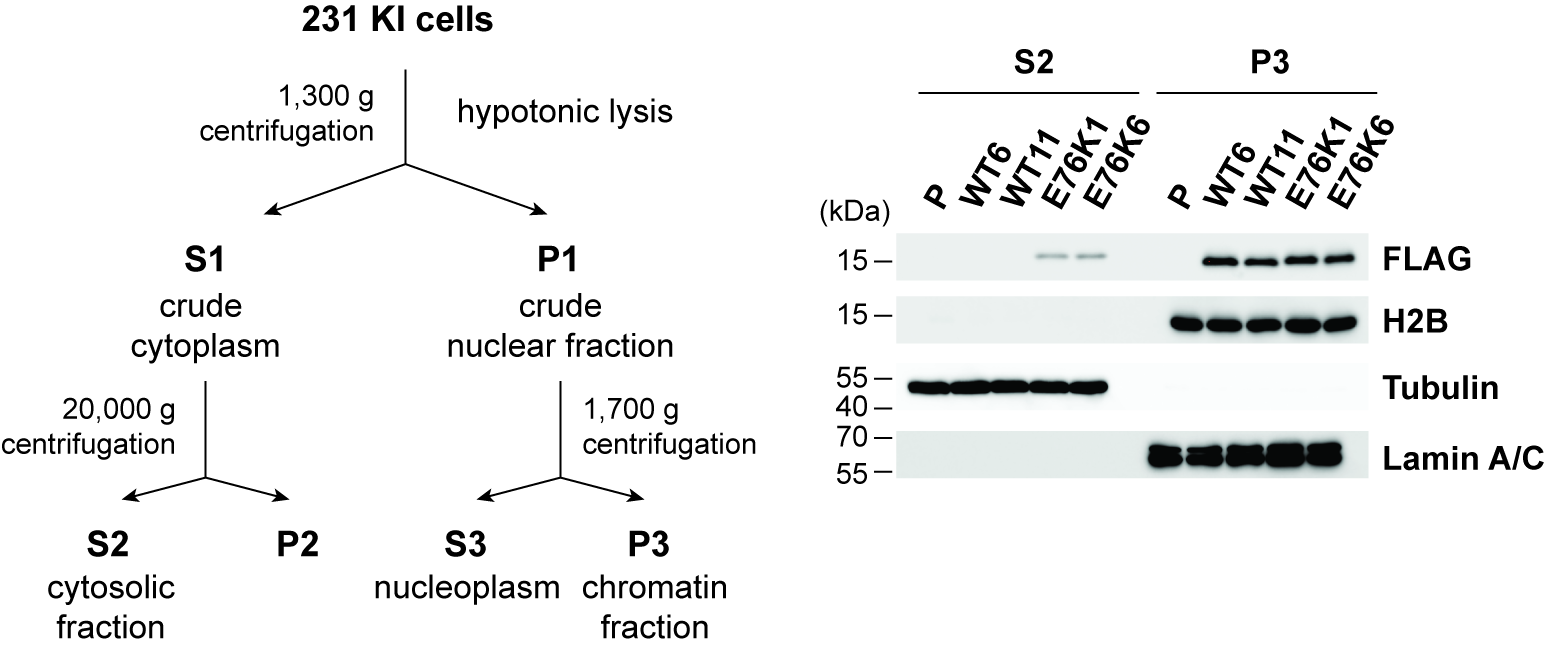


**H2BE76K expressed in the KI cells show chromatin localization defect.** Parental MDA-MB-231 and KI cell extracts were fractionated into S2 (cytosolic), S3 (soluble nuclear) and P3 (chromatin) fractions. Left panel: Cell fractionation scheme. Right panel: Immunoblot analysis of FLAG-tagged H2B proteins in cytosolic and chromatin fractions using indicated antibodies. Tubulin and Lamin A/C act as fractionation controls.

**Figure S5**


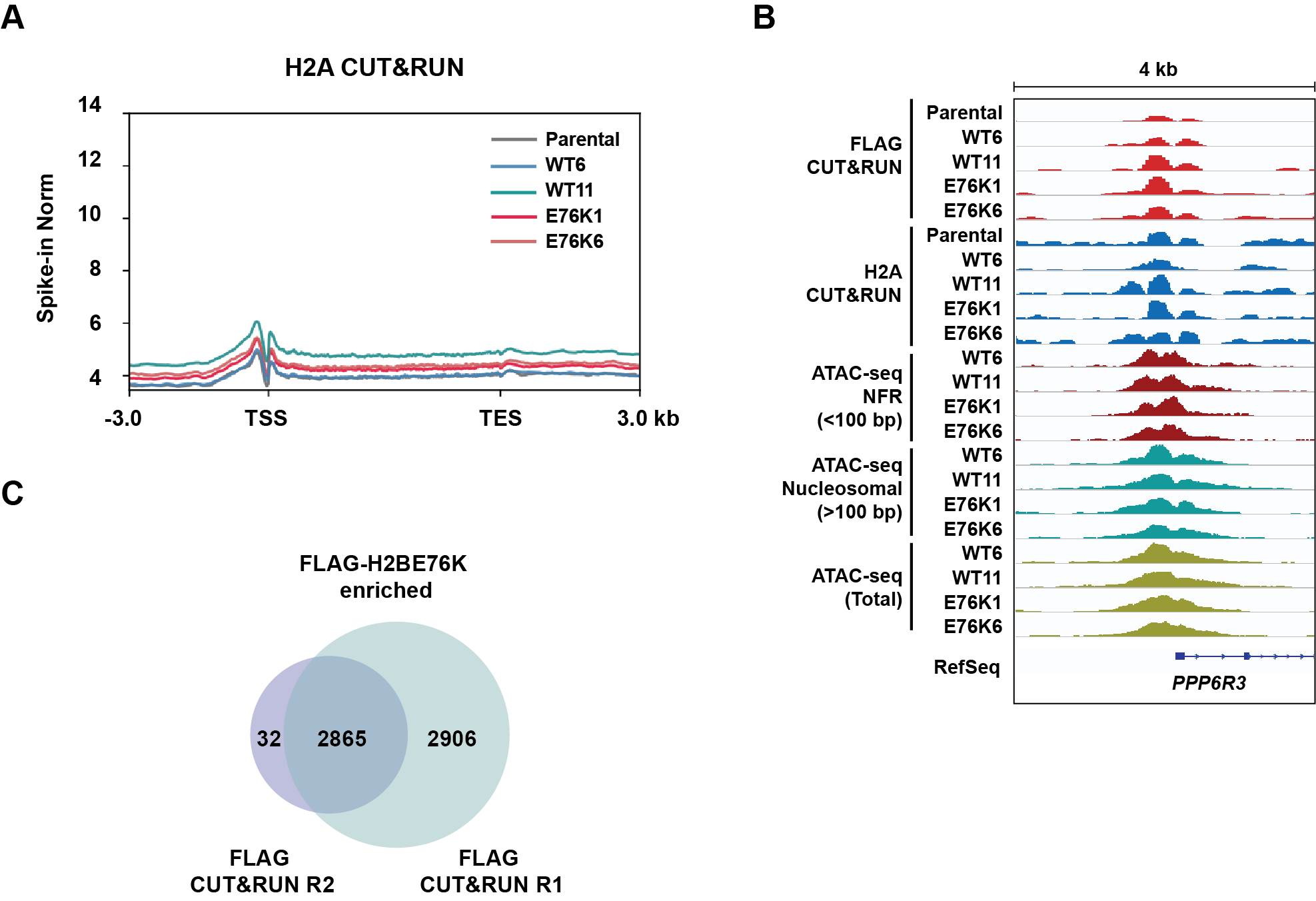


**H2BE76K is differentially enriched in more than 2000 genes**. **(A)** Metaplot of average H2A CUT&RUN enrichment on gene bodies in parental, WT and E76K cell lines from 3kb upstream of TSS to 3kb downstream of TES. Data are represented as yeast spike-in normalized reads. (**B)** FLAG CUT&RUN showed good correlation with regulatory nucleosomes, based on H2A CUT&RUN and ATAC-seq nucleosomal signal (>100 bp). IGV snapshot of FLAG, H2A CUT&RUN and ATAC-seq tracks of the TSS (±2 kb) region of *PPP6R3*. Total ATAC-seq reads (composite) were filtered based on fragment length. Reads shorter than 100 bp were considered nucleosomal free regions (NFR) and reads longer than 100 bp were considered nucleosomal signals. (**C)** Differential occupancy analysis of two independent FLAG CUT&RUN experiments produced consistent results and showed significant H2BE76K enrichment (BH adjusted *p* < 0.05, log_2_ fold enrichment > 0.5) on more than 2000 genes. Venn diagram showing the overlap of FLAG-H2BE76K enriched genes from two experiments.

# Figure S6


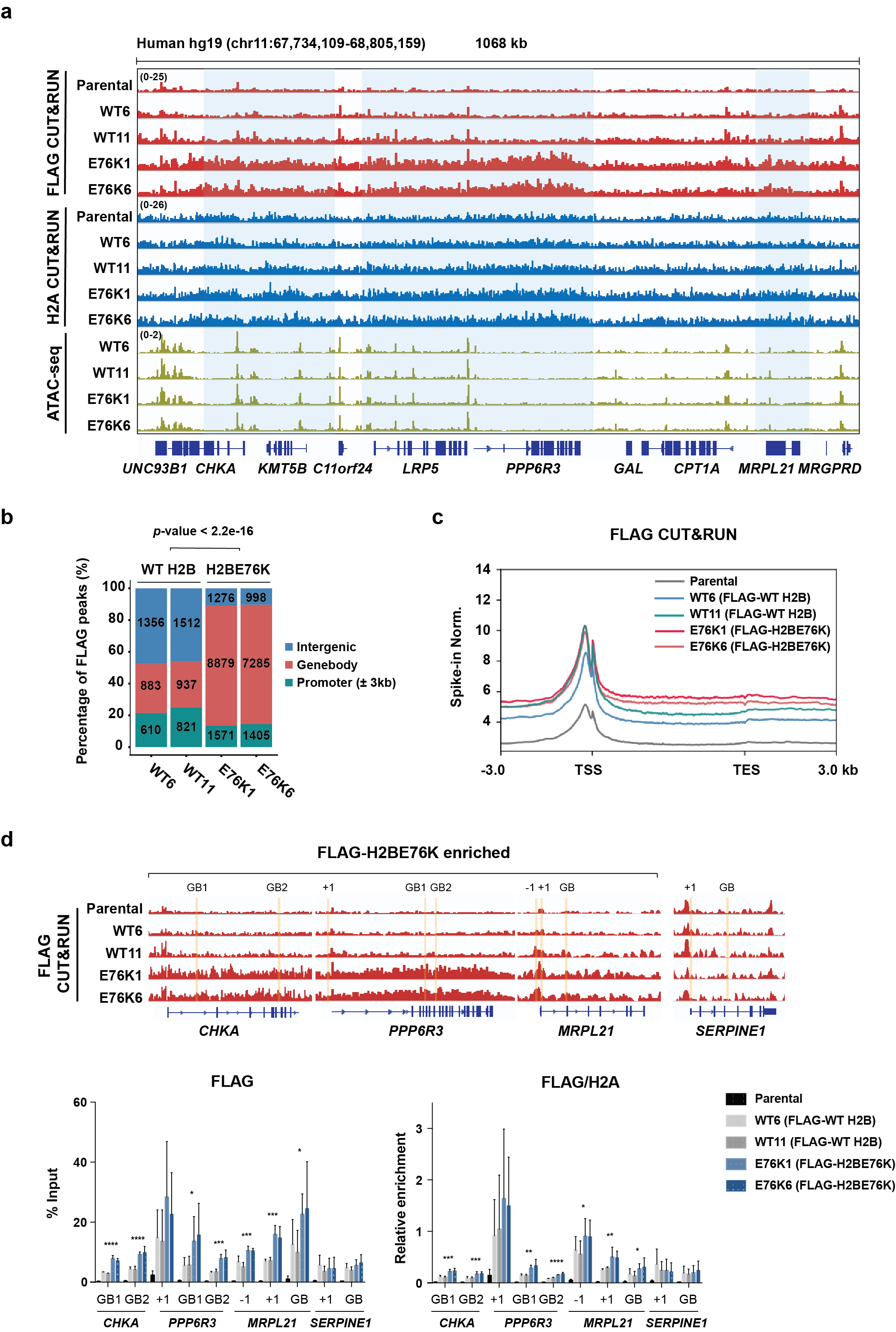


**ChIP-qPCR validation of H2BE76K enrichment.** Anti-FLAG immunoprecipitated DNA was analyzed for FLAG enrichment on H2BE76K-enriched genes, *CHKA*, *PPP6R3* and *MRPL21* in WT and E76K cell lines. *SERPINE1* acts as a negative control. IGV tracks of FLAG and H2A CUT&RUN show annotated PCR amplified regions. Multiple primer pairs targeting different regions including -1 and +1 nucleosomes and gene bodies (GB) were used for ChIP-qPCR analyses. Results from three independent experiments are shown (mean ± SD, **p* ≤ 0.05, ***p* ≤ 0.01, ****p* ≤ 0.001, *****p* ≤ 0.0001). *p*-values were calculated using one-sided *t*-test between WT and H2BE76K samples.

# Figure S7


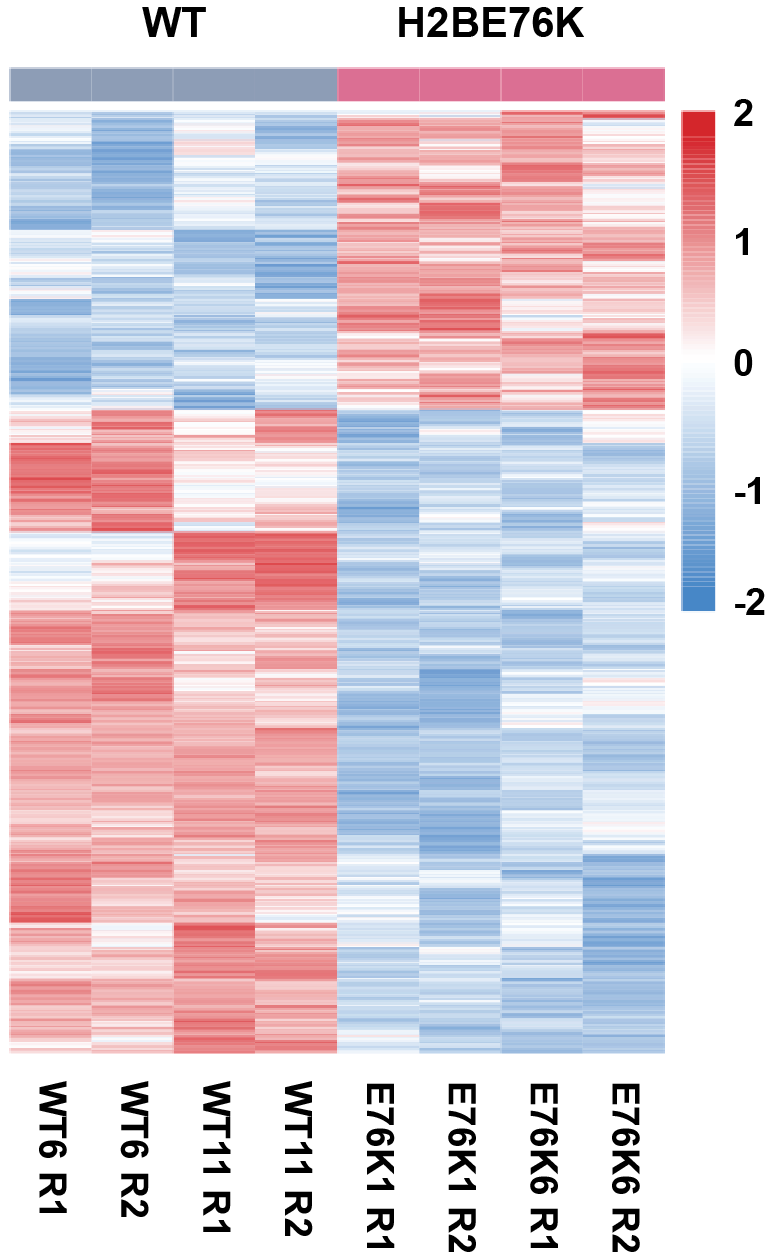


**H2BE76K cells display altered transcriptome**. RNA-seq heatmap showing the expression Z-score of significant differentially expressed genes (BH adjusted *p* < 0.05, absolute log_2_FC > 0.25) between WT and H2BE76K mutant cells. The heatmap shows 228 upregulated and 493 downregulated genes (H2BE76K vs. WT). Consistent results were obtained from two independent RNA-seq experiments.

**Figure S8**

**
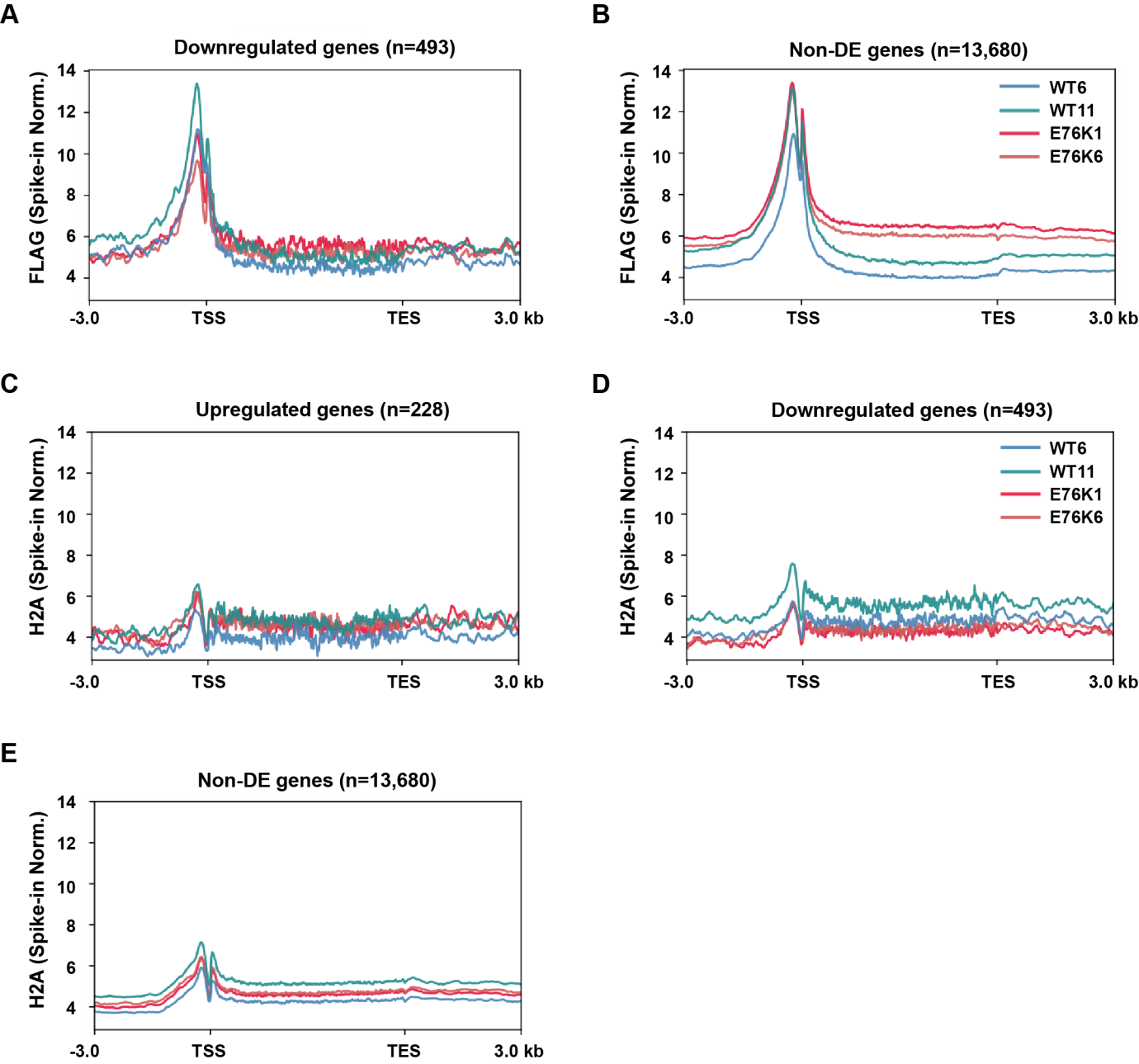
**

**Upregulated genes in H2BE76K mutant cells are H2BE76K enriched. (A-B)** Metaplots showing average FLAG CUT&RUN-seq signals on downregulated and non-DE genes. (**C-E)** Metaplot of average H2A CUT&RUN-seq signals on upregulated, downregulated and non-DE genes. The gene groups were categorized according to their differential expression between H2BE76K mutant and WT cells. Data are represented as yeast spike-in normalized reads.

# Figure S9

**
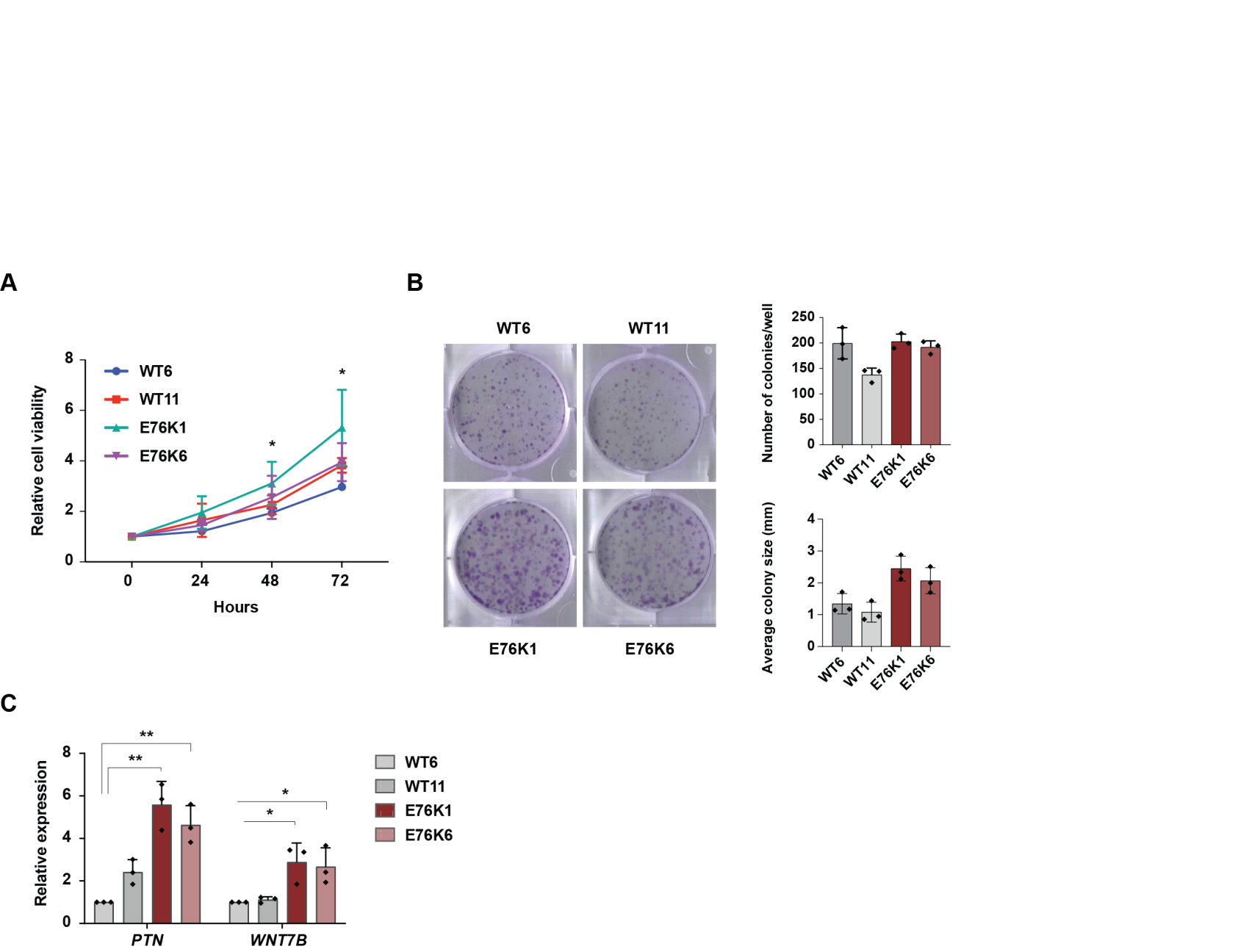
**

**H2BE76K mutant cells form bigger colonies. (A)** WT and H2BE76K mutant cells show comparable proliferation rates. Cell viability was measured using CCK-8 assay. Relative absorbance at 450 nm was measured at indicated time points after cell seeding and normalized to the absorbance at 0 h. Results from three independent experiments are shown (mean ± SD). *p*-values were calculated using one-sided *t*-test compared with WT for each time point (**p* ≤ 0.05). **(B)** Colony formation assay with WT and H2BE76K KI cell lines. Number and size of colonies per well were calculated from average values from triplicate wells of each independent experiment. Mean ± SD from three independent experiments are shown. **(C)** mRNA expression of *PTN* and *WNT7B* in WT and H2BE76K cell lines. Results from three independent RT-qPCR experiments are shown (mean ± SD, **p* ≤ 0.05, ***p* ≤ 0.01). *p*-values were calculated using the one-sided *t*-test.

# Figure S10


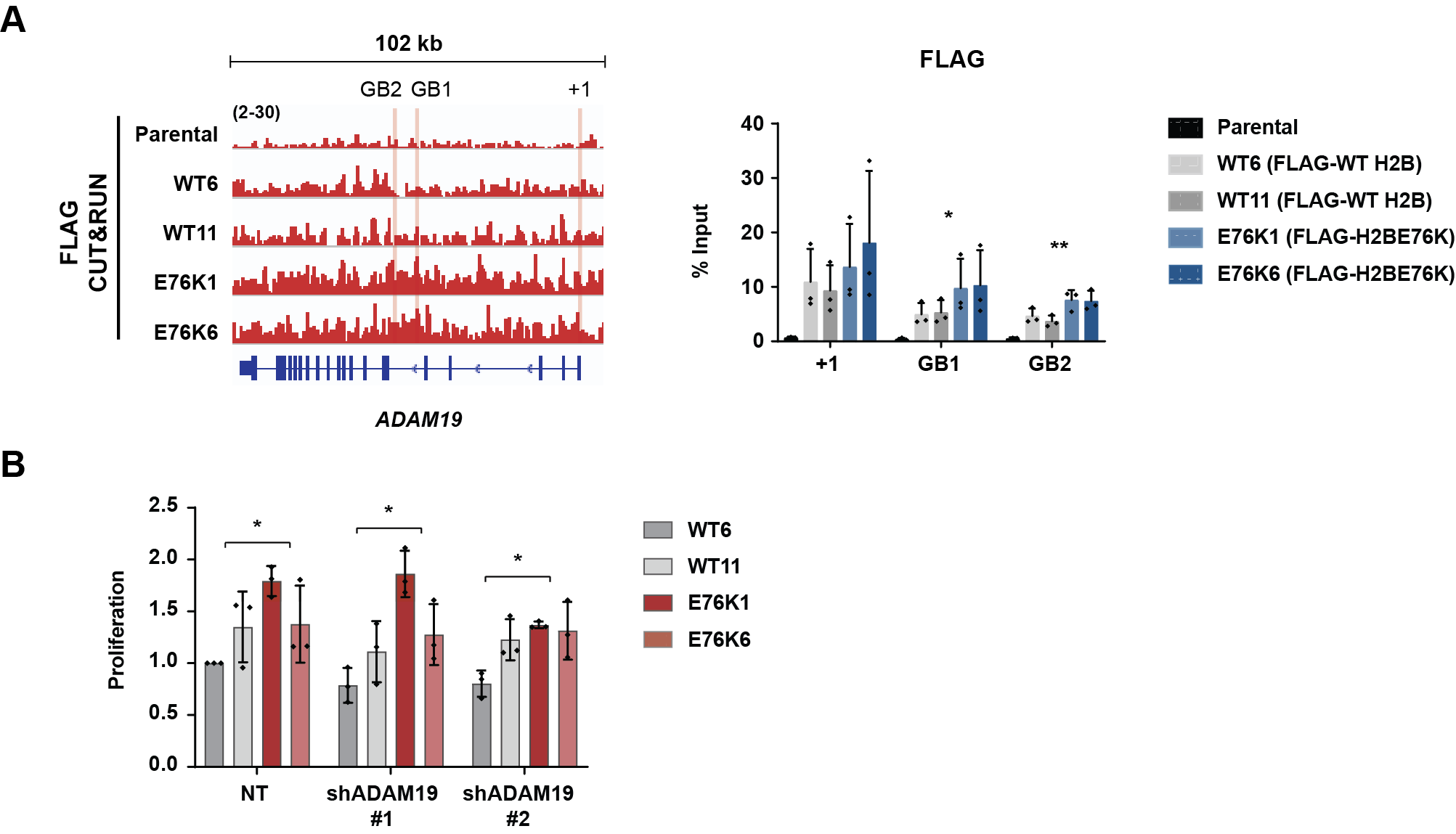


**(A) ChIP-qPCR validation of FLAG-H2BE76K enrichment on *ADAM19***. IGV tracks of FLAG CUT&RUN show PCR amplified regions in the +1 nucleosome and gene body (GB). Mean ± SD from three independent experiments are shown. *p*-values were calculated using one-sided *t*-test compared with WT (**p* ≤ 0.05, ***p* ≤ 0.01). **(B)** ***ADAM19* depletion does not affect cell proliferation**. Proliferation was calculated using CCK-8 assay. Y-axis shows the relative absorbance at 450 nm 72 hours after cell seeding normalized to the absorbance at 0 h. Results are shown as mean ± SD from three independent experiments. *p*-values were calculated using one-sided *t*-test compared with WT (**p* ≤ 0.05).

# Figure S11

***ADAM19* is highly expressed in various cancers**. Boxplots showing *ADAM19* expression in different cancer types. Expression data was downloaded from the TCGA database. *p*-values were calculated by two-tailed Student’s *t*-test.

# Figure S12


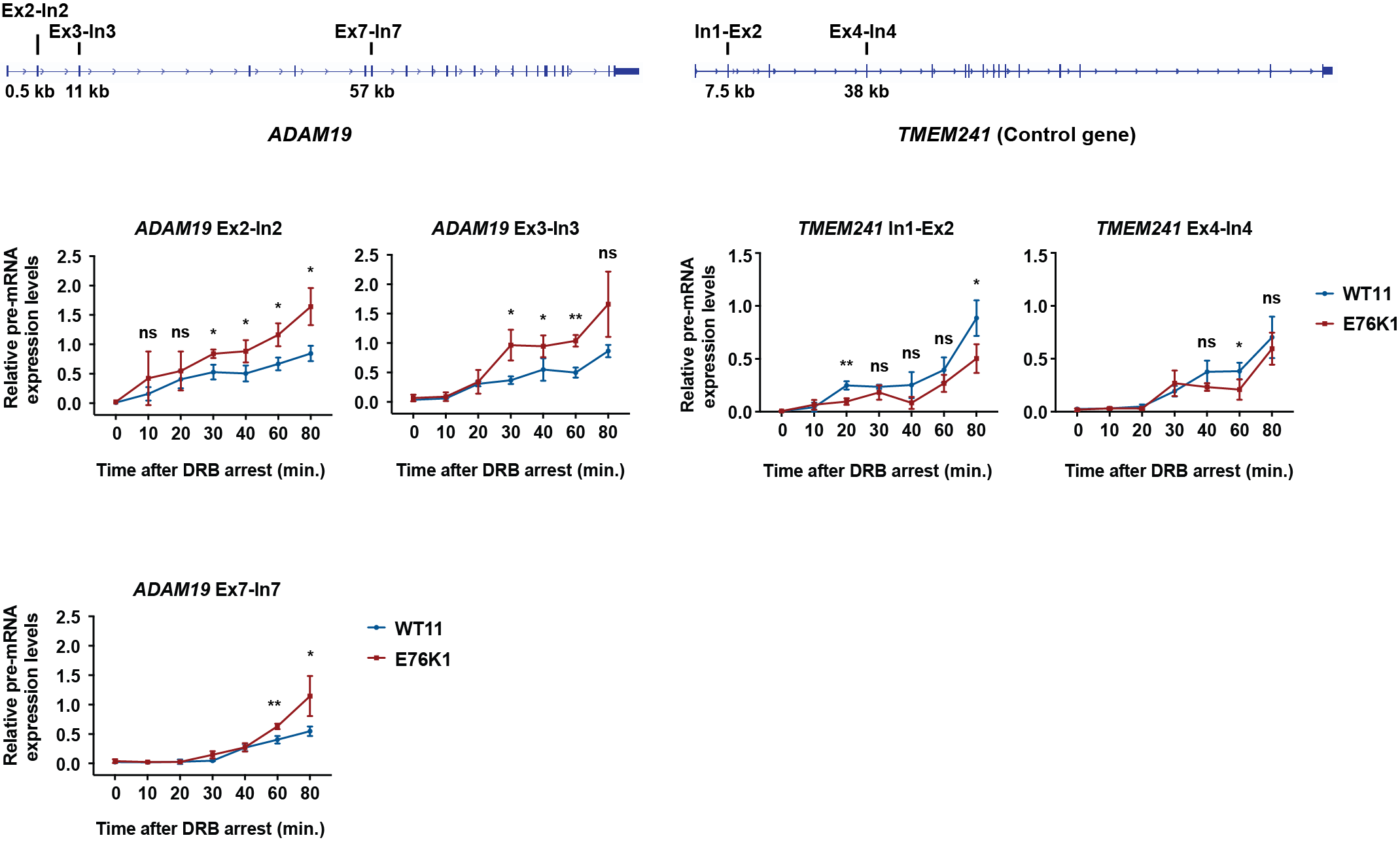


***ADAM19* expression recovered more efficiently in the H2BE76K cells after DRB washout**. The experiments shown in **Fig. 5** was independently repeated with another WT/ H2BE76K mutant pair. Pre-mRNA expression levels of *ADAM19* and the control gene, *TMEM241*, were monitored at the indicated time points after release from DRB inhibition. Results are shown as mean ± SD from three independent experiments. *p*-values were calculated using one-sided t-test at each time point (**p* ≤ 0.05, ***p* ≤ 0.01, n.s. not significant.).

# Figure S13


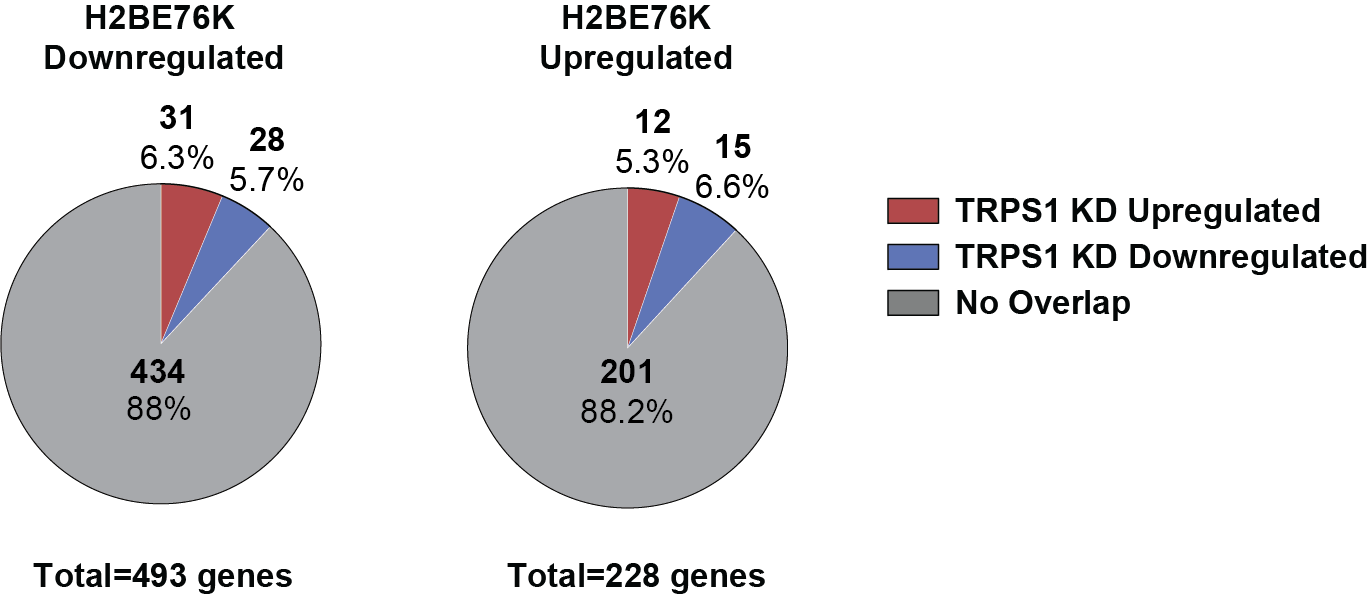


**The upregulation of the H2BE76K-enriched TRPS1 in H2BE76K mutant cells may induce indirect gene expression changes.** Piecharts show the common genes differentially expressed (left: downregulated, right: upregulated) in H2BE76K mutant cells and in TRPS1-depleted HCC3153 and SUM159 cells.

**Table S1** **H2BE76K and H2BE76Q mutations were found in breast and lung cancers.** Glutamic acid (E) at 76 was found mutated to either lysine (K) or glutamine (Q) in some of the 17 genes encoding histone H2B in breast and lung carcinomas. Cancer patient samples data was obtained from cBioPortal^1^ and COSMIC^2^.

| **Gene Symbol** | **Sample ID** | **Cancer types** | **Mutation** | **Allele frequency** |
| --- | --- | --- | --- | --- |
| *HIST1H2BG* | BR-M-037 | Breast Invasive Ductal Carcinoma | E76K |  |
| *HIST1H2BH* | TCGA-AC-A23H | Breast Invasive Ductal Carcinoma | E76K | 0.55 |
| *HIST1H2BH* | TCGA-AC-A6IW | Breast Invasive Ductal Carcinoma | E76Q | 0.11 |
| *HIST1H2BD* | TCGA-OL-A5RW | Breast Invasive Ductal Carcinoma | E76Q | 0.69 |
| *HIST1H2BF* | TCGA-A2-A0EY | Invasive Breast Carcinoma | E76K | 0.14 |
| *HIST3H2BB* | TCGA-E9-A3X8-01 | Breast Invasive Lobular Carcinoma | E76K | 0.09 |
| *HIST1H2BC* | PD6722a | Breast Carcinoma | E76K |  |
| *HIST1H2BD* | PD4120a | Breast Carcinoma | E76K |  |
| *HIST1H2BF* | DN12100 | Breast Carcinoma | E76K |  |
| *HIST1H2BC* | TCGA-33-6738 | Lung Squamous Cell Carcinoma | E76K | 0.19 |
| *HIST1H2BE* | TCGA-33-AASJ | Lung Squamous Cell Carcinoma | E76K | 0.23 |
| *HIST1H2BF* | TCGA-34-2600 | Lung Squamous Cell Carcinoma | E76K | 0.13 |
| *HIST1H2BO* | TCGA-66-2759 | Lung Squamous Cell Carcinoma | E76K | 0.38 |
| *HIST1H2BH* | TCGA-66-2778 | Lung Squamous Cell Carcinoma | E76K | 0.04 |
| *HIST1H2BH* | TCGA-39-5034 | Lung Squamous Cell Carcinoma | E76K | 0.44 |
| *HIST1H2BD* | TCGA-66-2773 | Lung Squamous Cell Carcinoma | E76Q | 0.18 |
| *HIST1H2BC* | TCGA-77-A5GB | Lung Squamous Cell Carcinoma | E76Q | 0.18 |
| *HIST1H2BG* | TCGA-60-2720 | Lung Squamous Cell Carcinoma | E76Q | 0.17 |
| *HIST1H2BI* | LUAD-RT-S01769 | Lung Adenocarcinoma | E76K | 0.24 |
| *HIST1H2BI* | LUAD-RT-S01699 | Lung Adenocarcinoma | E76K | 0.09 |
| *HIST1H2BH* | TCGA-55-7907 | Lung Adenocarcinoma | E76K | 0.3 |
|  |  |  |  | **Average= 0.24** |

**Table S2 78 upregulated and H2BE76K-enriched genes.** List of genes that are upregulated in the H2BE76K mutant cells (BH adjusted *p* < 0.05, absolute log_2_FC > 0.25) and H2BE76K enriched (BH adjusted *p* < 0.05, log_2_ fold enrichment > 0.5).

| **Gene** | **RNA-seq log_2_ FC** | **RNA-seq adjusted *p*** | **FLAG CUT&RUN log_2_ fold enrichment** | **FLAG CUT&RUN adjusted *p*** |
| --- | --- | --- | --- | --- |
| *COCH* | 2.75 | 7.66E-10 | 0.77 | 7.42E-05 |
| *FAM133A* | 3.86 | 1.21E-09 | 0.572 | 4.90E-02 |
| *TNIK* | 0.954 | 2.30E-09 | 0.645 | 5.88E-10 |
| *DPF3* | 4.69 | 1.47E-08 | 0.525 | 2.31E-04 |
| *CDH4* | 0.475 | 1.58E-07 | 0.523 | 4.69E-08 |
| *MRPL21* | 0.613 | 1.79E-06 | 0.988 | 5.10E-07 |
| *NTF3* | 6.61 | 3.17E-06 | 0.815 | 3.24E-06 |
| *TGFBRAP1* | 0.428 | 1.86E-05 | 0.98 | 1.79E-15 |
| *SYNJ2* | 0.428 | 7.40E-05 | 0.584 | 1.22E-05 |
| *PFAS* | 0.393 | 9.87E-05 | 0.578 | 2.70E-03 |
| *MYEOV* | 0.821 | 1.30E-04 | 0.565 | 6.00E-05 |
| *DCAF4* | 0.925 | 1.93E-04 | 0.805 | 1.48E-06 |
| *ZFHX4* | 0.678 | 3.42E-04 | 0.857 | 2.14E-12 |
| *GXYLT1* | 0.445 | 3.42E-04 | 0.931 | 8.46E-12 |
| *SPTLC3* | 0.777 | 3.42E-04 | 0.965 | 1.16E-08 |
| *SHANK2* | 1.04 | 4.06E-04 | 0.504 | 4.16E-07 |
| *MEGF9* | 0.516 | 5.92E-04 | 0.861 | 3.75E-09 |
| *ADAM19* | 0.738 | 8.59E-04 | 0.502 | 7.74E-05 |
| *CEP250* | 0.416 | 9.83E-04 | 0.731 | 1.27E-08 |
| *ANKRD27* | 0.341 | 1.01E-03 | 0.865 | 2.10E-12 |
| *PPP6R3* | 0.255 | 1.27E-03 | 1.56 | 7.52E-44 |
| *TFPI* | 0.444 | 1.58E-03 | 0.626 | 5.00E-06 |
| *GALNT7* | 0.267 | 2.16E-03 | 0.739 | 1.10E-10 |
| *ZMIZ1* | 0.291 | 2.94E-03 | 0.613 | 9.02E-09 |
| *ADGRA3* | 0.431 | 2.94E-03 | 0.818 | 1.22E-10 |
| *CFDP1* | 0.539 | 3.28E-03 | 0.692 | 5.42E-09 |
| *MYO5A* | 0.443 | 3.47E-03 | 0.901 | 8.69E-16 |
| *DOCK11* | 0.819 | 3.59E-03 | 0.574 | 2.24E-07 |
| *NPM1* | 0.369 | 3.90E-03 | 0.855 | 6.35E-05 |
| *MAGI1* | 0.352 | 3.93E-03 | 1.16 | 1.27E-33 |
| *ABCC4* | 0.341 | 4.39E-03 | 0.788 | 1.38E-11 |
| *ACSS1* | 0.735 | 4.62E-03 | 0.618 | 2.30E-03 |
| *FLG* | 0.764 | 4.64E-03 | 0.749 | 9.72E-04 |
| *SH3BGRL* | 0.494 | 4.73E-03 | 1.06 | 2.21E-16 |
| *CAMK4* | 0.647 | 5.10E-03 | 0.71 | 1.05E-10 |
| *ZNF37A* | 0.343 | 5.41E-03 | 0.609 | 9.79E-04 |
| *LAS1L* | 0.363 | 6.09E-03 | 0.93 | 3.45E-06 |
| *LRP5* | 0.251 | 7.58E-03 | 0.91 | 1.68E-17 |
| *GNPTAB* | 0.542 | 8.85E-03 | 0.676 | 2.78E-08 |
| *ZNF480* | 0.415 | 9.09E-03 | 0.738 | 9.38E-05 |
| *C3orf14* | 0.486 | 9.56E-03 | 0.51 | 3.40E-02 |
| *LARS* | 0.534 | 1.01E-02 | 1.04 | 5.84E-17 |
| *CST7* | 0.919 | 1.04E-02 | 0.627 | 3.91E-02 |
| *C4orf19* | 0.531 | 1.15E-02 | 0.744 | 9.42E-12 |
| *PTCH1* | 0.421 | 1.21E-02 | 0.572 | 3.04E-06 |
| *SRGAP1* | 0.269 | 1.28E-02 | 0.809 | 1.15E-14 |
| *C20orf194* | 0.429 | 1.28E-02 | 1.26 | 2.79E-04 |
| *ARHGAP26* | 0.538 | 1.41E-02 | 0.77 | 1.84E-11 |
| *CCDC112* | 0.686 | 1.42E-02 | 0.918 | 7.55E-09 |
| *SLC36A1* | 0.545 | 1.42E-02 | 0.674 | 2.16E-06 |
| *SLC35F3* | 0.52 | 1.51E-02 | 0.937 | 3.46E-22 |
| *RALGPS2* | 0.289 | 1.54E-02 | 0.961 | 2.97E-20 |
| *DTD1* | 0.624 | 1.55E-02 | 1.43 | 1.30E-04 |
| *SMYD3* | 0.343 | 1.56E-02 | 1.13 | 6.93E-31 |
| *GNPDA1* | 0.485 | 1.63E-02 | 0.566 | 2.33E-02 |
| *CMC1* | 0.632 | 1.71E-02 | 0.971 | 1.13E-15 |
| *HARS* | 0.333 | 1.71E-02 | 0.698 | 3.66E-04 |
| *ITGBL1* | 0.826 | 1.76E-02 | 0.591 | 3.56E-07 |
| *IDH3B* | 0.422 | 1.81E-02 | 0.938 | 1.02E-02 |
| *OSBPL6* | 0.65 | 2.16E-02 | 0.58 | 7.03E-08 |
| *RIN2* | 0.402 | 2.34E-02 | 0.811 | 4.60E-02 |
| *TRPS1* | 0.371 | 2.54E-02 | 0.753 | 2.16E-12 |
| *NDUFS6* | 0.489 | 2.68E-02 | 0.562 | 5.23E-03 |
| *HIPK2* | 0.482 | 2.79E-02 | 0.958 | 5.02E-22 |
| *RSL24D1* | 0.313 | 2.80E-02 | 1.17 | 4.83E-08 |
| *IL17RA* | 0.343 | 3.10E-02 | 0.588 | 3.07E-03 |
| *PAK1* | 0.411 | 3.19E-02 | 0.759 | 1.50E-11 |
| *FRA10AC1* | 0.557 | 3.21E-02 | 0.652 | 2.79E-05 |
| *VPS16* | 0.453 | 3.22E-02 | 0.58 | 1.08E-02 |
| *HSPA9* | 0.336 | 3.36E-02 | 1.04 | 1.38E-07 |
| *PCDHGC3* | 1.34 | 3.54E-02 | 0.64 | 1.10E-04 |
| *ZNF512* | 0.271 | 3.73E-02 | 0.801 | 2.96E-09 |
| *DIAPH2* | 0.275 | 3.90E-02 | 1.17 | 1.27E-30 |
| *RSU1* | 0.283 | 4.05E-02 | 0.98 | 1.03E-20 |
| *EBPL* | 0.397 | 4.06E-02 | 0.682 | 7.60E-04 |
| *TMX4* | 0.703 | 4.40E-02 | 0.716 | 3.08E-03 |
| *ADAM22* | 0.469 | 4.58E-02 | 0.702 | 6.47E-12 |
| *CTSC* | 0.367 | 4.87E-02 | 0.774 | 6.67E-08 |

**Table S3 sgRNA sequences for CRISPR/Cas9 knock-in**

| **sgRNA** | **Sequence** |
| --- | --- |
| 5' sgRNA | GGAGCAGATTTAGCCGGGTCGGG |
| 3' sgRNA | TCTTTTGGGTGGACTCCGGCCGG |

**Table S4 Primers used to genotype CRISPR-Cas9 KI clones**

| **Genotype** | **Sequence (5’-3’)** |
| --- | --- |
| Insertion | F- AGTAACTTCCAATCAGACAG |
|  | R- AGCGTCTATACTCACACGCAAA |
| 5' integration | F- GTCTCGTTATCAGGGTGGTC |
|  | R- GAAGAACTCGTCAAGAAGGC |
| 3' integration | F- CAAGGATTACAAAGACGATG |
|  | R- CCTCCTATTTCTGTGATCTC |

**Table S5 shRNAs used in this study**

| **Gene** | **shRNA** | **Sequence** |
| --- | --- | --- |
| *ADAM19* | sh1 | CCGGCCCTACCAACTTCTACCAGATCTCGAGATCTGGTAGAAGTTGGTAGGGTTTTTG |
|  | sh2 | CCGGCCATTATACTTCAAGTGGTAACTCGAGTTACCACTTGAAGTATAATGGTTTTTG |

**Table S6 Primers used for RT-qPCR**

| **Gene** | **Sequence (5'-3')** |
| --- | --- |
| *PIK3CG* | F- AGAGTTCCATATGATCCTGG |
|  | R- AATCTCTCCACTGCTGCCTG |
| *c-MYC* | F- TGCCTTGGTTCATCTGGGTC |
|  | R- GCTTAGGAGTGCTTGGGACA |
| *CDKN1A* | F- CTGGAAGGGGAAGGGACACACAAGA |
|  | R- AGGAAGGTCGCTGGACGATTTGAGG |
| *EPHA2* | F- TGTGCCAGGCAGGCTACG |
|  | R- CTCCAAGCAGGGGCTCTCA |
| *ACTIN* | F- TGACGTGGACATCCGCAAAG |
|  | R- CTGGAAGGTGGACAGCGAGG |
| *ADAM19* | F- AGAGTGTGGGTCCTGTGGTA |
|  | R- AACTGAACTGTTGCCTCAGC |

**Table S7 Primers used for FLAG ChIP-qPCR**

| **Gene** | **Region** | **Sequence (5'-3')** |
| --- | --- | --- |
| *CHKA* | GB1 | F- AGAATTCTACTCCAGCACGG |
|  |  | R- AACATTCCTGCAGTACACAGG |
|  | GB2 | F- ACCTGAACTACTGTGACTAGG |
|  |  | R- ATCCCATGCAATTGCACATTG |
| *PPP6R3* | +1 | F- TGATGGTGTCGGTGAGCG |
|  |  | R- ATTACCTGGAGGTGCCCA |
|  | GB1 | F- GCAGGTTAGATGGGAGAGCG |
|  |  | R- GAGAGGAGCCTCCATTGCAT |
|  | GB2 | F- GTCTAGTTCAGCTCCAGATC |
|  |  | R- AGATGTGGCTGGAATGGAAC |
| *MRPL21* | -1 | F- CTCATCACTTCCTGGGCAG |
|  |  | R- GCCCAGGAGTGTAAGCTTAGT |
|  | +1 | F- CAGGCTACTAACTGAAGGAATC |
|  |  | R- CAGGTGAGACCTGGGATATG |
|  | GB | F- GGTAGATATGAAGTGCTCTGTG |
|  |  | R- GCAGTGGTTCATACCATGTAC |
| *SERPINE1* | +1 | F- AAGAGCGCTGTCAAGAAGAC |
|  |  | R- CTCCTACCTGAAGTTCTCAG |
|  | GB | F- TGCTAGTGTGATTACCTGGG |
|  |  | R- CTCATCCTCTTAACCTCAAC |
| *ADAM19* | +1 | F- CTGGATGGACAAGTAAGTGG |
|  |  | R- AAGGCTACTCCCAGGTCTCC |
|  | GB1 | F- TCATAGTGGCTGCAGATTATAG |
|  |  | R- CATTTGTACGTTGGGGACAATA |
|  | GB2 | F- CCTAAAGAACTGCTTTGAAGAG |
|  |  | R- CATGATTGGAGAAATAGGTAGG |

**Table S8 Primers used for Pol II ChIP-qPCR**

| **Gene** | **Region** | **Sequence (5’-3’)** |
| --- | --- | --- |
| *ADAM19* | -250 bp TSS | F- AGGAGGAGGGATTTGTGGTG |
|  |  | R- GAATCACCTCTGAGTGTCAC |
|  | +200 bp TSS | F- CTGGATGGACAAGTAAGTGG |
|  |  | R- AAGGCTACTCCCAGGTCTCC |
|  | GB | F- ACCTCAGTTGTCAACTCCTG |
|  |  | R- CCAAGCTGCAGCATGAACTTA |
| *TMEM241* | -250 bp TSS | F- CGCAGTTCACAATACCCCAT |
|  |  | R- GTCATTTGCCAAAGCGCTACA |
|  | +350 bp TSS | F- CCTGGAGATGTACCGTGCTTT |
|  |  | R- GATGGAGAGCGAACCTCCTC |
|  | GB | F- GTAGGGTAGGTAAATTTCAAGAC |
|  |  | R- GTCTTTGATTATATGGTTTGTCAG |

**Table S9 Primers used for DRB-RT qPCR**

| **Gene** | **Region** | **Sequence (5'-3')** |
| --- | --- | --- |
| *TMEM241* | In1-Ex2 | F- GTAGGGTAGGTAAATTTCAAGAC |
|  |  | R- GTCTTTGATTATATGGTTTGTCAG |
|  | Ex3-In4 | F- GCCCTTATTGAGCTGAGGCA |
|  |  | R- GGGTCCAGAGCATTGTCCAG |
| *ADAM19* | Ex2-In2 | F- ACCTCAGTTGTCAACTCCTG |
|  |  | R- CCAAGCTGCAGCATGAACTTA |
|  | Ex3-In3 | F- CTTCCAGAACAGCGACACCT |
|  |  | R- GGCGAGAACTGATCCTGGAC |
|  | Ex7-In7 | F- AGTCTGGAGCCTGGAATGGA |
|  |  | R- GAGCTTTACCTCGTGGCTGA |
